# Heterologous Production of Glycine Betaine Using *Synechocystis* sp. PCC 6803-Based Chassis Lacking Native Compatible Solutes

**DOI:** 10.3389/fbioe.2021.821075

**Published:** 2022-01-07

**Authors:** Eunice A. Ferreira, Catarina C. Pacheco, João S. Rodrigues, Filipe Pinto, Pedro Lamosa, David Fuente, Javier Urchueguía, Paula Tamagnini

**Affiliations:** ^1^ I3S—Instituto de Investigação e Inovação em Saúde, Universidade do Porto, Porto, Portugal; ^2^ IBMC—Instituto de Biologia Molecular e Celular, Universidade do Porto, Porto, Portugal; ^3^ ICBAS—Instituto de Ciências Biomédicas Abel Salazar, Universidade do Porto, Porto, Portugal; ^4^ Departamento de Biologia, Faculdade de Ciências, Universidade do Porto, Porto, Portugal; ^5^ Instituto de Tecnologia Química e Biológica António Xavier, ITQB NOVA, Oeiras, Portugal; ^6^ Instituto de Aplicaciones de las Tecnologías de la Información y de las Comunicaciones Avanzadas, Universitat Politècnica de València, València, Spain

**Keywords:** compatible solutes, cyanobacteria, glycine betaine, glucosylglycerol, salt stress, sucrose, *Synechocystis*, synthetic biology

## Abstract

Among compatible solutes, glycine betaine has various applications in the fields of nutrition, pharmaceuticals, and cosmetics. Currently, this compound can be extracted from sugar beet plants or obtained by chemical synthesis, resulting in low yields or high carbon footprint, respectively. Hence, in this work we aimed at exploring the production of glycine betaine using the unicellular cyanobacterium *Synechocystis* sp. PCC 6803 as a photoautotrophic chassis. *Synechocystis* mutants lacking the native compatible solutes sucrose or/and glucosylglycerol—∆*sps*, ∆*ggpS*, and ∆*sps*∆*ggpS*—were generated and characterized. Under salt stress conditions, the growth was impaired and accumulation of glycogen decreased by ∼50% whereas the production of compatible solutes and extracellular polymeric substances (capsular and released ones) increased with salinity. These mutants were used as chassis for the implementation of a synthetic device based on the metabolic pathway described for the halophilic cyanobacterium *Aphanothece halophytica* for the production of the compatible solute glycine betaine. Transcription of ORFs comprising the device was shown to be stable and insulated from *Synechocystis’* native regulatory network. Production of glycine betaine was achieved in all chassis tested, and was shown to increase with salinity. The introduction of the glycine betaine synthetic device into the ∆*ggpS* background improved its growth and enabled survival under 5% NaCl, which was not observed in the absence of the device. The maximum glycine betaine production [64.29 µmol/gDW (1.89 µmol/mg protein)] was reached in the ∆*ggpS* chassis grown under 3% NaCl. Taking into consideration this production under seawater-like salinity, and the identification of main key players involved in the carbon fluxes, this work paves the way for a feasible production of this, or other compatible solutes, using optimized *Synechocystis* chassis in a pilot-scale.

## Introduction

Microorganisms can cope with environmental stresses such as temperature, salinity or drought via the production of compatible solutes (CS)—low-molecular weight organic compounds highly soluble in water that can accumulate intracellularly up to molar concentrations, without interfering with the cell metabolism (Klähn and Hagemann, 2011). CS belong to different chemical classes including sugars (e.g., sucrose, trehalose), polyols (e.g., glycerol, sorbitol), heterosides (e.g., glucosylglycerol, floridoside), and amino acids or their derivatives (e.g., proline, glutamate, glycine betaine, ectoine) (Kirsch et al., 2019). Glycine betaine (or *N*,*N*,*N*-trimethylglycine) is an ubiquitous solute that can be found in bacteria, plants and mammals, being mostly synthesized by the two-step oxidation of choline to betaine aldehyde and subsequently to glycine betaine (Landfald and Strøm, 1986; Grossman and Hebert, 1989; Rathinasabapathi et al., 1997). Later on, the synthesis of glycine betaine *via* a three-step methylation of glycine was described in extremely halophilic bacteria (Nyyssola et al., 2000; Waditee et al., 2003). This biosynthetic pathway involves two *N*-methyltransferases: the glycine-sarcosine-*N*-methyltransferase (GSMT) that catalyzes the methylation of glycine and sarcosine, and the dimethylglycine-*N*-methyltransferase (DMT) that converts dimethylglycine to glycine betaine. This CS has a strong stabilizing effect on biomolecules, by maintaining their structure and function (Guinn et al., 2011; Stadmiller et al., 2017), and thus conferring drought-, osmo- and thermo-protection to cells (Caldas et al., 1999; Holmström et al., 2000; Cleland et al., 2004; You et al., 2019). Moreover, glycine betaine plays an important physiological role as methyl group donor with beneficial stress-mitigating effects in humans (Lever and Slow, 2010; Day and Kempson, 2016), and up-regulating antioxidant defense systems in plants (Rady et al., 2018; Sun et al., 2020). Due to these interesting properties, glycine betaine is a value-added compound with applications in human nutrition, animal feed, agriculture, pharmaceuticals, and cosmetics (Eklund et al., 2005; Lever and Slow, 2010; Nsimba et al., 2010; Cholewa et al., 2014; Day and Kempson, 2016; Dikilitas et al., 2020). Most of the commercially available glycine betaine is extracted from sugar beets (*Beta vulgaris*) (Heikkila et al., 1982), resulting in relatively low yields and rendering this organic production an expensive process. Alternatively, glycine betaine can be produced by chemical synthesis, but this process, although cheaper, has a high environmental impact increasing the carbon footprint (DuPont, 2015; Kar et al., 2015). A more sustainable and cost-effective production is highly desirable, and thus cyanobacteria emerge as promising chassis for the production of compatible solutes and other products of interest. Their photoautotrophic metabolism enables the sequestration and conversion of atmospheric CO_2_ into organic compounds using sunlight and water as energy and electron sources, respectively (Knoll, 2008; Ananya and Ahmad, 2014). Therefore, they are being increasingly studied to be used as solar-powered cell factories for many biotechnological applications including the production of e.g., alcohols, alkanes, hydrogen, sugars, and terpenoids (Hays and Ducat, 2015; Lindblad, 2018; Sadvakasova et al., 2020; Wang et al., 2020; Rodrigues and Lindberg, 2021). Among cyanobacteria, the unicellular *Synechocystis* sp. PCC 6803 (hereafter *Synechocystis*) is the best studied strain, and the vast array of data generated over the past decades allowed the construction of genome-scale metabolic models to predict system’s behavior (Montagud et al., 2010; Montagud et al., 2011; Joshi et al., 2017; Gopalakrishnan et al., 2018). Moreover, various molecular and synthetic biology tools are now available for the genetic manipulation and engineering of this particular cyanobacterial strain (Huang et al., 2010; Heidorn et al., 2011; Pinto et al., 2015; Pacheco et al., 2021). *Synechocystis* is a freshwater strain and thus moderately halotolerant, relying on the biosynthesis of the compatible solutes sucrose, glutamate and glucosylglycerol to maintain the osmotic pressure under stress conditions (Klähn and Hagemann, 2011; Iijima et al., 2020).

In this study, *Synechocystis* knockout mutants in the biosynthetic pathways producing the main native compatible solutes sucrose or/and glucosylglycerol were generated to serve as chassis for the production of value-added compounds. The genome-scale metabolic model *i*Syn811 was used to simulate the production rates of different heterologous CS and the highest rate was predicted for glycine betaine. As a proof-of-concept, we explored the production of this compatible solute using our *Synechocystis*-based chassis. For this purpose, a synthetic device based on the biosynthetic gene cluster from the halophilic cyanobacterium *Aphanothece halophytica* was constructed and introduced in the different chassis. Besides showing the production of glycine betaine and validating the functionality of the synthetic device, the characterization of the strains contributes to a better understanding of the mechanisms used by the cells to maintain homeostasis and cope with different levels of salinity.

## Materials and Methods

### Reagents and Enzymes

The media components and other reagents were obtained from Fisher Scientific (United States), Merck (Germany) or Sigma Aldrich (United States), and noble agar from Difco (United States). All DNA-modifying enzymes and polymerases were purchased from Thermo Fisher Scientific (United States) and Promega (United States), and standard molecular biology kits were obtained from NZY Tech (Portugal). The Sanger sequencing and oligo synthesis services were provided by STAB VIDA, Lda (Portugal).

### Organisms and Culture Conditions

Wild-type and mutants of the unicellular, non-motile cyanobacterium *Synechocystis* sp. PCC 6803 substrain GT-Kazusa (Kanesaki et al., 2012; Trautmann et al., 2012) (obtained from the Pasteur Culture Collection, France) were maintained in Erlenmeyer flasks batch cultures with BG11 medium (Stanier et al., 1971) at 30°C with orbital shaking (150 rpm) under a 12 h light/12 h dark regimen. Light intensity was 25 μE/m^2^/s in all experiments and Cosine-corrected irradiance was measured using a Dual Solar/Electric Quantum Meter (Spectrum Technologies, Inc., United States). For solid BG11, the medium was supplemented with 1.5% (wt/vol) noble agar, 0.3% (wt/vol) sodium thiosulfate and 10 mM TES-KOH buffer, pH 8.2 (Stanier et al., 1971). For the selection and maintenance of mutants, BG11 medium was supplemented with chloramphenicol (Cm, 10–20 µg/ml). For cloning purposes, *E. coli* strains DH5α and XL1-blue were used. Cells were grown at 37°C in LB medium (Sambrook and Russel, 2001), supplemented with kanamycin (Km, 50 µg/ml) or Cm (34 µg/ml).

### DNA and RNA Extraction

Cyanobacterial genomic DNA (gDNA) extraction was performed according to the procedure described previously (Tamagnini et al., 1997). For RNA extraction, 50 ml of *Synechocystis* culture at OD_730_ ≈ 1 was centrifuged for 10 min at 4,470 *g*; cell pellets were treated with RNAprotect Bacteria Reagent (Qiagen, Germany) according to instructions, and stored at −80°C. RNA was extracted using the TRIzol^®^ Reagent (Ambion) according to the method described previously (Leitão et al., 2006) with the following adaptations: the cells were disrupted using a FastPrep^®^-24 (MP Biomedicals) in 2 cycles of 1 min at 4.0 m/s and the RNA samples were treated with 1 U of RQ1 RNase-Free DNase (Promega) according to manufacturer’s instructions.

### Glycine Betaine Device: Design, DNA Synthesis, and Assembly

The synthetic construction for the synthesis of glycine betaine (Ahbet) was designed based on *gsmt* (encoding the glycine-sarcosine-*N*-methyltransferase) and *dmt* (encoding the dimethylglycine-*N*-methyltransferase) Open Reading Frames (ORFs) from the cyanobacterium *Aphanothece halophytica*, and the *metX* (*sll0927*, encoding *S*-adenosyl-methionine synthase) ORF from *Synechocystis*. All the ORF sequences were codon optimized for *Synechocystis* using the Gene Designer 2.0 software (DNA 2.0, Inc., United States), restriction sites incompatible with the BioBrick^™^ standard RFC [10] were eliminated and double stop codons included. Each ORF is preceded by the BioBrick^™^ (BB) ribosome binding site (RBS) BBa_B0030 and the double terminator BBa_B0015 was included after the *metX* ORF. In addition, the synthetic construction is flanked by the prefix and suffix sequences of the BB RFC [10] standard. All the BB sequences were retrieved from the Registry of Standard Biological Parts (parts.igem.org). Subsequently, the sequence of the glycine betaine synthetic construction flanked by the BB prefix, the double terminator and BB suffix was synthesized and cloned into *Sma*I digested pBluescript II SK(-) (Epoch Life Science, Inc., United States).

To construct the glycine betaine device, the synthesized Ahbet construct was assembled with the synthetic promoter P_*trc.x.lacO*,_ previously characterized in *Synechocystis* (Ferreira et al., 2018). For this purpose, the Ahbet was PCR-amplified from the plasmid pBSK with the pUC primers (Additional file 1: Supplementary Table S4), using Phusion high-fidelity DNA polymerase, according to the manufacturer’s instructions. The PCR product was purified using NZYGelpure kit, digested with *Xba*I and *Pst*I and cloned downstream of P_*trc.x.lacO*_ in the pJ201 plasmid (digested with *Spe*I and *Pst*I, and dephosphorylated). The generated P_*trc.x.lacO*_::Ahbet device was excised from the pJ201 plasmid with *Xba*I and *Spe*I, and transferred to pSEVA351 shuttle vector (Silva-Rocha et al., 2013), digested with *Xba*I. The pSEVA351 was obtained from the “Standard European Vector Architecture” repository and is comprised by the broad-host-range replicon RSF1010 and the chloramphenicol antibiotic marker. The assembly and transfer of the synthetic device was confirmed by PCR, restriction analysis and Sanger sequencing.

### Construction of Integrative Plasmids for the Generation of CS Mutants

The construction of integrative plasmids for the knockout of *ggpS* (glucosylglycerol-phosphate synthase) and *sps* (sucrose-phosphate synthase) genes was performed as described previously (Pinto et al., 2012). Briefly, the plasmids were based on pGEM-T^®^ Easy (Promega, United States) and contain the *Synechocystis* chromosomal regions flanking the *ggpS* or the *sps* gene. The 5′- and 3′-flanking regions were amplified from the cyanobacterium’s genome using *Pfu* DNA polymerase and the primer pairs 5-O/5-I and 3-O/3-I (Additional file 1: Supplementary Table S4), respectively. Subsequently, the purified PCR fragments were fused by Overlap Extension PCR using primers 5-O/3-O and 80 ng of each amplicon. The resulting product was purified and cloned into the vector pGEM-T^®^ Easy, according to the manufacturer’s instructions, originating the pGDggpS, and the pGDsps plasmids (Table 1). A selection cassette, containing the *nptII* gene (conferring resistance to neomycin and kanamycin) and the *sacB* gene (conferring sensitivity to sucrose), was PCR amplified from the plasmid pK18mobsacB (Schafer et al., 1994) with specific primers (Additional file 1: Supplementary Table S4). The amplicon was then cloned into the *Age*I/*Sma*I restriction site of pGDggpS or pGDsps plasmids, generating the pGDggpS.KS and the pGDsps.KS plasmids, respectively (Table 1). All constructs were confirmed by sequencing.

**TABLE 1:** List of plasmids used to transform *Synechocystis.*

Designation	Plasmid	Description	Reference/Source
P_*trc.x.lacO*_::Ahbet	pSEVA351	Ahbet synthetic construction under the control of the P_*trc.x.lacO*_ promoter	This study
pGDggpS	pGEM-T® Easy	pGEM-T easy vector containing the two regions for double homologous recombination targeting the *ggpS locus*	Ferreira et al. (2018)
pGDggpS.KS	pGEM-T® Easy	pGEM-T easy vector containing the *nptII* and *sacB* genes flanked by the two regions for double homologous recombination targeting the *ggpS locus*	This study
pGDsps	pGEM-T® Easy	pGEM-T easy vector containing the two regions for double homologous recombination targeting the *sps locus*	This study
pGDsps.KS	pGEM-T® Easy	pGEM-T easy vector containing the *nptII* and *sacB* genes flanked by the two regions for double homologous recombination targeting the *sps locus*	This study

### Generation of *Synechocystis* CS Knockout Mutants

*Synechocystis* was transformed based on the protocol described by Williams (1988) with modifications. *Synechocystis* cultures were grown under standard conditions to an OD_730_ ≈ 0.5. Cells were harvested by centrifugation at 3,850 *g* for 10 min; and then resuspended in BG11 to a final OD_730_ ≈ 2.5. A 500 μL aliquot of these cells was used (per transformation) and incubated with purified pGDggpS.KS or pGDsps.KS plasmids, at a final DNA concentration of 20 µg/ml, for 5 h in light at 30°C. Cells were then spread onto Immobilon^™^-NC membranes (0.45 µm pore size, 82 mm, Millipore, United States) resting on solid BG11 plates, incubated at 25°C under low light, and transferred to selective solid BG11 plates supplemented with 10 µg/mL km after 24 h. Transformants were observed after 1–2 weeks. For complete segregation, Km-resistant colonies were streaked on BG11 plates with increasing Km concentrations (up to 500 µg/ml), and finally transferred into liquid medium. Mutants were then tested for sucrose sensitivity and confirmed by PCR and Southern blot (for details see below). Subsequently, to remove the selection markers from the insertion mutants, cells were transformed as described above with the pGDggpS or the pGDsps plasmids, and the mutants were selected on solid BG11 containing 10% (wt/vol) sucrose. These mutants were also screened for Km-sensitivity. The double mutant ∆*sps*∆*ggpS* was generated by deleting the *ggpS* gene from the ∆*sps* background following the abovementioned protocol. The full segregation of the mutants was confirmed by PCR using GoTaq^®^ G2 Flexi DNA Polymerase, together with specific primers (Additional file 1: Supplementary Table S4), according to manufacturer’s instructions. Mutant segregation was also confirmed by Southern blots that were performed using 4 μg of genomic DNA of the wild-type and mutants, digested with *Mun*I (wild-type, ∆*sps.KS*, ∆*sps*, ∆*ggpS.KS*, and ∆*ggpS*), and *Ava*II (wild-type, ∆*sps*∆*ggpS.KS*, and ∆*sps*∆*ggpS*). The DNA fragments were separated by electrophoresis on a 1% (wt/vol) agarose gel and blotted onto Hybond^™^-N membrane (GE Healthcare, United States). Probes covering the 5′ flanking region of the *ggpS* or 3′ flanking region of the *sps* genes were amplified by PCR (using primers indicated in Additional file 1: Supplementary Table S4), and labeled using the DIG DNA labelling kit (Roche Diagnostics GmbH, Germany), according to the manufacturer’s instructions. Hybridization was performed overnight at 65°C, and digoxigenin-labelled probes were detected by chemiluminescence using CPD-star (Roche) in a Chemi Doc^™^ XRS^+^ Imager (Bio-Rad, United States).

### Introduction of the Glycine Betaine Synthetic Device Into *Synechocystis*

The pSEVA351 plasmid containing the synthetic device P_*trc.x.lacO*_::Ahbet (Table 1; sequence provided in Supplementary Datasheet S2) was introduced into *Synechocystis* by electroporation following the protocol described previously (Ferreira et al., 2018). The presence of the synthetic device was confirmed in *Synechocystis* transformants by PCR using specific primers (Additional file 1: Supplementary Table S4), as described by Ferreira et al. (2018).

### Growth Experiments

Pre-cultures of *Synechocystis* wild-type and mutants were inoculated in BG11 medium (supplemented with 10 μg/ml Cm, when appropriate) and grown in an orbital shaker (150 rpm), at 30°C under a 12 h light (25 μE/m^2^/s)/12 h dark regimen. The cultures were grown to an OD_730_ ≈ 2 and, subsequently, diluted in fresh BG11 medium without antibiotic to a final OD_730_ ≈ 0.5. Fifty milliliters of the dilution were transferred to 100 ml Erlenmeyer flasks without NaCl or containing 3, 5, or 7% (wt/vol) NaCl (510, 860, and 1,200 mM NaCl, respectively), previously sterilized. These cultures were maintained in the same conditions as the pre-cultures and growth was monitored for at least 16 days, by measuring the optical density at 730 nm (OD_730_) and determining the chlorophyll *a* (chl *a*) content as described by Meeks and Castenholz (1971). All the growth experiments included, at least, three biological replicates with technical duplicates.

### Total Carbohydrate Content, Released and Capsular Polysaccharides Measurements

Total carbohydrate content and RPS were determined as previously described (Mota et al., 2013). Briefly, 10 ml of culture samples were dialyzed (12–14 kDa molecular weight cutoff; CelluSepT4, Orange Scientific) against at least 10 volumes of distilled water, 3 or 5% (wt/vol) NaCl solutions (identical to the growth medium), for at least 24 h. One milliliter of the collected sample was used to spectrophotometrically quantify the total carbohydrate content by the phenol-sulfuric acid method (Dubois et al., 1956), whereas 5 ml of the dialyzed sample was centrifuged at 3,857 *g* for 10 min at RT, and the cell-free supernatant was used to determine the RPS. For CPS quantification, the procedure was performed as described previously (Pereira et al., 2019). Five milliliters of dialyzed cultures were centrifuged at 3,857 *g* for 10 min at RT, the cell pellet was resuspended in water and boiled for 15 min. After centrifugation as described previously, the cell-free supernatant was used for CPS measurement by the phenol-sulfuric acid method (Dubois et al., 1956). Total carbohydrate content, RPS and CPS were normalized by chl *a* content. All experiments included, at least, three biological replicates with technical triplicates.

### Glycogen Extraction and Quantification

Glycogen extraction was performed as described previously (Ernst et al., 1984). Ten milliliters of cell culture were collected 1 h after the transition between the dark and the light phase. Samples were centrifuged, and the cell pellets suspended in 100 μL of distilled water and 400 μL of 30% (wt/vol) KOH was added. The mixture was incubated at 100°C for 90 min and then quickly cooled on ice. Six hundred μL of ice-cold absolute ethanol were added, and the mixture was incubated on ice for 2 h. The mixture was centrifuged for 5 min at maximum speed and 4°C. The supernatant was discarded, and the isolated glycogen was washed three times with 500 μL of ice-cold absolute ethanol and dried at 60°C. Glycogen quantification was performed by the phenol-sulfuric acid method (Dubois et al., 1956), and normalized by chl *a* content. Experiments included, at least, three biological replicates with technical triplicates.

### Optical Microscopy

Cultures of *Synechocystis* wild-type (WT) and the ∆*sps* mutant were grown in BG11 or BG11 supplemented with 5% (wt/vol) NaCl as stated above (initial OD_730_ ≈ 0.5). Four days after inoculation, cells were stained with 0.5% (wt/vol) of Alcian Blue (in 3% (vol/vol) acetic acid) in 1:1 (culture:dye) ratio. This mixture was added to 10 μL of 1% (wt/vol) low-melting point agarose beds (dissolved in BG11 medium) and covered with a coverslip. The preparations were observed using the light microscope Olympus DP25 Camera software Cell B.

### Transcription Analysis by RT-qPCR

After extraction (for details see above), RNA concentration and purity (the ratios A_260_/A_280_ and A_260_/A_230_) were measured using a NanoDrop ND-1000 spectrophotometer (NanoDrop Technologies, Inc., United States). The quality and integrity of the RNA samples was also inspected in 1% (wt/vol) agarose gel performed by standard protocols using TAE buffer. The absence of genomic DNA contamination was checked by PCR, in reaction mixtures containing 0.5 U of GoTaq^®^ G2 Flexi DNA Polymerase, 1x Green GoTaq Flexi buffer, 200 µM of each dNTP, 1.5 mM MgCl_2_, 0.25 µM of each rnpB primer (Additional file 1: Supplementary Table S4), and 200 ng of total RNA. The PCR profile was: 5 min at 95°C followed by 25 cycles of 20 s at 95°C, 20 s at 56°C and 20 s at 72°C, and a final extension at 72°C for 5 min. The PCR reactions were run on 1% (wt/vol) agarose gel as described above. For cDNA synthesis, 1 µg of total RNA was transcribed with the iScript^™^ Reverse Transcription Supermix for RT-qPCR (Bio-Rad) in a final volume of 20 μL, following the manufacturer’s instructions. A control PCR was performed using 1 µL of cDNA as a template, the BD16S primers (Additional file 1: Supplementary Table S4), and the same reaction conditions and PCR program described above. Five-fold standard dilutions of the cDNAs were made (1/5, 1/25, 1/125, and 1/625) and stored at −20°C. RT-qPCRs were performed on Hard-Shell 384-Well PCR Plates (thin wall, skirted, clear/white) covered with Microseal^®^ B adhesive seal (Bio-Rad). The reactions (10 µL) were manually assembled and contained 0.125 μM of each primer (Additional file 1: Supplementary Table S4), 5 μL of iTaq^™^ Universal SYBR^®^ Green Supermix (Bio-Rad) and 1 μL of template cDNA (dilution 1/5). The PCR protocol used was: 3 min at 95°C followed by 45 cycles of 30 s at 95°C, 30 s at 56°C, and 30 s at 72°C. In the end, a melting curve analysis of the amplicons (10 s cycles between 55 and 95°C with a 0.5°C increment per cycle) was conducted. Standard dilutions of the cDNA were used to check the relative efficiency and quality of primers, and negative controls (no template cDNA) included (for more details on RT-qPCR parameters see Additional file 1: Supplementary Table S5). RT-qPCRs were performed with three biological replicates and technical triplicates of each cDNA sample in the CFX384 Touch^™^ Real-Time PCR Detection System (Bio-Rad). The data obtained were analyzed using the Bio-Rad CFX Maestro^™^ 1.1 software, implementing an efficiency-corrected delta Cq method (ΔCq). This method was used since the target genes *gsmt*, *dmt*, and *metX* were validated as reference genes using the reference gene selection tool available in the Maestro^™^ software. For this reason, the relative expression of the targets is represented instead of the usual relative normalized expression. Statistical analysis was performed using a one-way ANOVA using the same software, and tests were considered significant if *p* < 0.05. The amplicon sizes were checked by agarose gel electrophoresis, and the DNA sequence was confirmed by Sanger sequencing. These experiments were compliant with the MIQE guidelines (Bustin et al., 2009), to promote the effort for experimental consistency and transparency, and to increase the reliability and integrity of the results obtained.

### Compatible Solutes Quantification

Cultures of *Synechocystis* wild-type (WT), the ∆*sps*, ∆*ggpS*, and ∆*sps*∆*ggpS* mutants and the strains harboring the GB device (WT, ∆*ggpS*, and ∆*sps*∆*ggpS* backgrounds) were grown in BG11 or BG11 supplemented with 3% or 5% (wt/vol) NaCl, as described above, at an initial OD_730_ ≈ 0.5. The quantification of the CS—sucrose, glutamate, glucosylglycerol, and glycine betaine—was performed using 500 ml culture (distributed in 50 ml cultures in 100 ml Erlenmeyer flasks). Four days after inoculation, cells were harvested by centrifugation at 4,470 *g* for 10 min at room temperature (RT). In the case of the strains harboring the GB device, the extracellular medium was stored at −80°C, for further lyophilization and CS extraction. Cells were washed using 100 ml of cold distilled water, 3 or 5% (wt/vol) NaCl solutions (identical to the growth medium). Centrifugation was repeated and the cell pellets were resuspended in 50 ml of the respective solutions. From this suspension, a 0.5 ml aliquot was centrifuged and stored at −20°C to be used later for protein quantification. The remaining cell suspension was centrifuged at 4°C and the cell pellet was stored at −20°C. Ethanol-chloroform extraction of the CS was performed as described in Ferreira et al. (2018) with adaptations. Briefly, cell pellets or lyophilized extracellular medium were suspended in 25 ml of 80% (vol/vol) ethanol and subsequently transferred to a 100 ml round flask containing a magnetic stir bar. The flask was connected to a coil condenser (circulating cold water) and heated at 100°C with stirring, for 10 min. The suspension was transferred to a 50 ml tube and centrifuged at 4,000 *g* for 10 min at RT. The supernatant was stored and the pellet resuspended in 20 ml of 80% (vol/vol) ethanol for a new extraction process. The remainder protocol was performed as described in Santos et al. (2006). Detection, identification and quantification of CS was performed by proton NMR. To that effect, freeze-dried extracts were dissolved in 1 ml of D_2_O and a known amount of sodium formate was added to serve as an internal concentration standard. Spectra were acquired at 25°C on a Bruker Avance III 800 spectrometer (Bruker, Rheinstetten, Germany) working at a proton operating frequency of 800.33 MHz, equipped with a 5 mm, three channel, inverse detection cryoprobe TCI-z H&F/C/N with pulse-field gradients. A 3 s soft pulse was applied before the excitation pulse, to pre-saturate the water signal. Spectra were acquired under fully relaxed conditions (flip angle 60°; repetition delay of 60 s) so that the area of the NMR signals was proportional to the amount of the different protons in the sample. Integration of the signals was performed using the tools available in the TopSpin software (Bruker, Rheinstetten, Germany) version 3.6.2. The concentration of CS was expressed as µmol per mg of protein. Protein extracts were obtained by sonication as described by Pinto et al. (2015), and protein quantification was performed using the Bio-Rad Protein Assay. For cell dry weight (DW) determinations, 40 ml of culture at OD_730_ ≈ 1.0 (or equivalent) was centrifuged at 3,857 *g* for 10 min at RT. Then, the cell pellet was dried at 60°C for 48 h. Experiments included, at least, three biological replicates.

### *In silico* Analysis of CS Production

The genome-scale metabolic model of *Synechocystis*—*i*Syn811 (Montagud et al., 2011) –, was updated to include all the information required for the simulations. The manual curation process started with the addition of the final reaction in the synthesis of sucrose (“spp: H_2_O+ sucrose 6-phosphate → phosphate + sucrose”), and also the metabolic precursors and the three reactions required for the synthesis of the heterologous CS, glycine betaine (“GSMT1: *S*-adenosyl-L-methionine + glycine ↔ *S*-adenosyl-L-homocysteine + sarcosine,” “GSMT2: *S*-adenosyl-L-methionine + sarcosine ↔ *S*-adenosyl-L-homocysteine + *N,N*-dimethylglycine,” and “DMT: *S*-adenosyl-L-methionine + *N*,*N*-dimethylglycine ↔ *S*-adenosyl-L-homocysteine + *N*,*N*,*N*-trimethylglycine”). In this process, the nomenclature was corrected and standardized (e.g., “glycerone” to “dihydroxy-acetone” or “GDP-mannose” to “GDP-D-mannose”), and the reversibility of some reactions changed (e.g., “sn-glycerol-3-phosphate → dihydroxy-acetone phosphate” to “sn-glycerol-3-phosphate ↔ dihydroxy-acetone phosphate”). Flux balance analysis (Orth et al., 2010) was performed to the *i*Syn811 genome-scale metabolic reconstruction of *Synechocystis* for the production assessment of four different CS: three heterologous (glycine betaine, ectoine, and mannosylglycerate), and three native (glucosylglycerol, glutamate and sucrose). The MATLAB software, COBRA Toolbox v3.0 (Heirendt et al., 2019) was used for quantitative prediction of cellular and multicellular biochemical networks with constraint-based modelling. Simulations were constrained to match an autotrophic specific growth rate of 0.09/h, which corresponds to a light input of 0.8 mE/gDW/h and to a net carbon flux of 3.4 mmol/gDW/h into the cell, with CO_2_ as carbon source. The description of the *i*Syn811 model and further information on the simulation procedure are available in Montagud et al. (2010).

### Statistical Analysis

The statistical analysis was performed by means of one- or two-way ANOVAs, using GraphPad Prism v6.01 (GraphPad Software Inc., United States).

## Results

### Generation of *Synechocystis* Mutants Deficient in the Synthesis of Native Compatible Solutes

The sustainable production of heterologous compatible solutes using *Synechocystis* as a chassis was envisioned in this work. The starting step was the generation of mutants deficient in the production of one or both of the main native compatible solutes, sucrose, or glucosylglycerol (GG). For this purpose, the genes encoding the enzymes involved in the first step of sucrose or/and GG synthesis (*sps* and *ggpS*, respectively), were knockout by double homologous recombination generating the *Synechocystis* markerless mutants Δ*sps*, Δ*ggpS*, and Δ*sps*Δ*ggpS* (for details see *Materials*). The complete segregation of the mutants was confirmed by PCR and Southern blot (Additional file 1: Supplementary Figures S1, S2).

### Effect of NaCl on the Growth of *Synechocystis* Wild-Type and the CS Deficient Mutants

**FIGURE 1:**
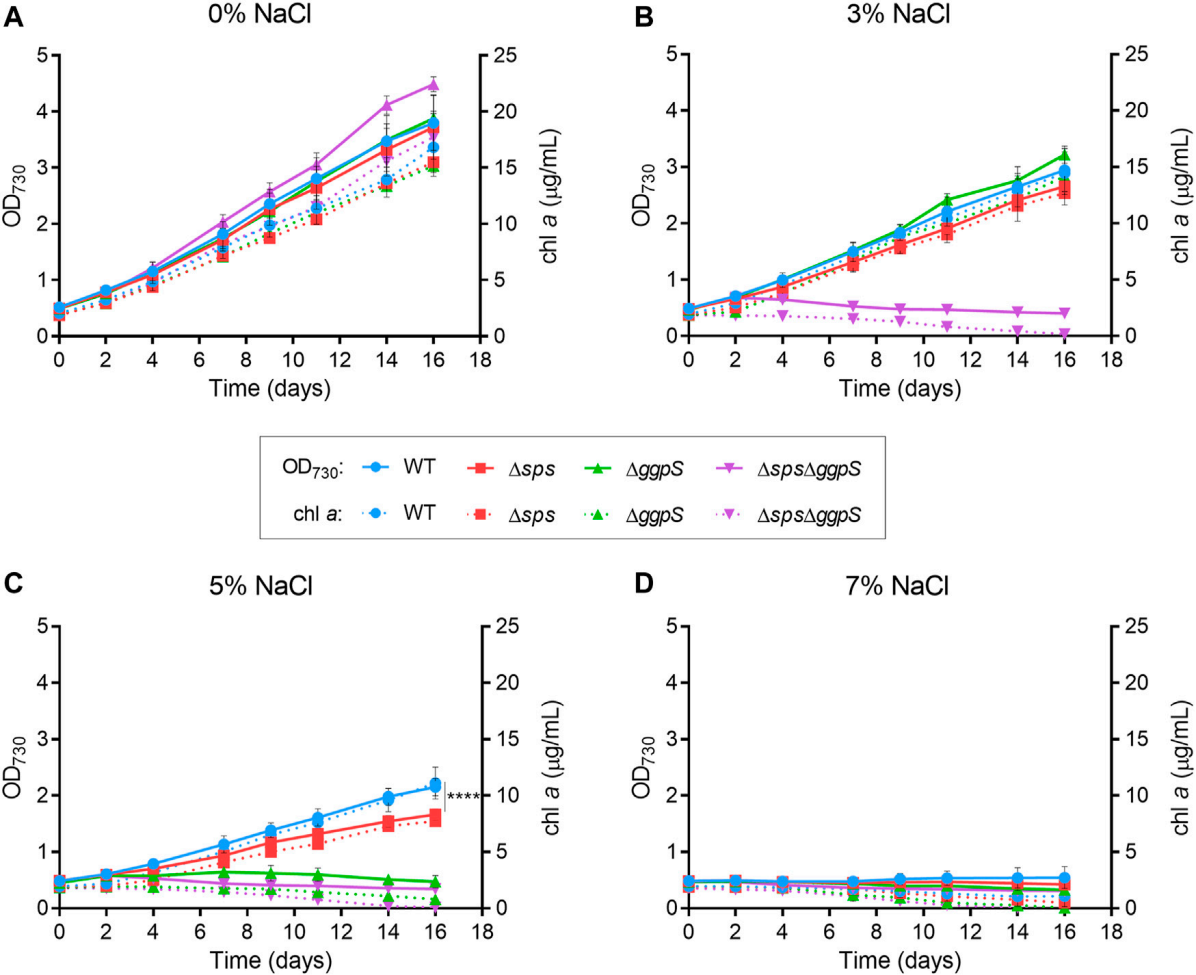
Growth curves of *Synechocystis* wild-type (WT) and ∆*sps*, ∆*ggpS* and ∆*sps*∆*ggpS* mutants in BG11 **(A)** or BG11 supplemented with 3% **(B)**, 5% **(C)** or 7% **(D)** (wt/vol) NaCl. Cultures were grown at 30°C with orbital shaking (150 rpm) under a 12 h light (25 μE/m^2^/s)/12 h dark regimen. Growth was monitored by measuring optical density at 730 nm (OD_730_), and chlorophyll *a* (chl *a*) (full and dotted lines, respectively). Error bars correspond to standard deviations from at least three biological replicates with technical duplicates. Statistical analysis was performed using a two-way ANOVA, and a significant difference in terms of OD_730_ is identified by **** (*p* ≤ 0.0001).

The growth of the CS deficient mutants under different salinities was analyzed. *Synechocystis* WT and mutants Δ*sps*, Δ*ggpS*, and Δ*sps*Δ*ggpS* were grown in standard BG11 medium or in BG11 supplemented with 3, 5, and 7% (wt/vol) NaCl, corresponding to 510, 860, and 1,200 mM, respectively. The growth was monitored by measuring the OD_730_ and chlorophyll *a* (chl *a*) content (Figure 1). In the absence of NaCl, the three mutants exhibited growth similar to the WT (Figure 1A), indicating that the synthesis of sucrose and/or GG is nonessential under standard growth conditions. Nonetheless, challenging the cells with 3% NaCl had clear detrimental effects, with a ∼23% growth decrease observed for the WT and CS single mutants Δ*sps* and Δ*ggpS* (Figure 1B; Additional file 1: Supplementary Table S1). The inactivation of both pathways in the Δ*sps*Δ*ggpS* mutant led to total growth arrest accompanied by a decline in chl *a* content (Figure 1B; purple lines and Additional file 1: Supplementary Table S1). A more pronounced impact was observed by increasing NaCl to 5%. The Δ*ggpS* could not grow in these conditions, while for the WT and Δ*sps*, a severe growth impairment (∼49%) was observed (Figure 1C; Additional file 1: Supplementary Table S1). The growth of the latter two strains was similar up to day 7 however, after this period, the growth of Δ*sps* slowed down and by day 16 there was a significant difference (*p* ≤ 0.0001) compared with the WT. The chl *a* content confirmed these observations (Figure 1C; red and blue lines). Further increase in the NaCl concentration to 7% (wt/vol) showed that none of the strains tested could withstand the stress imposed (Figure 1D; Additional file 1: Supplementary Table S1).

### Quantification of CS in *Synechocystis* Wild-Type and the CS Deficient Mutants

**FIGURE 2:**
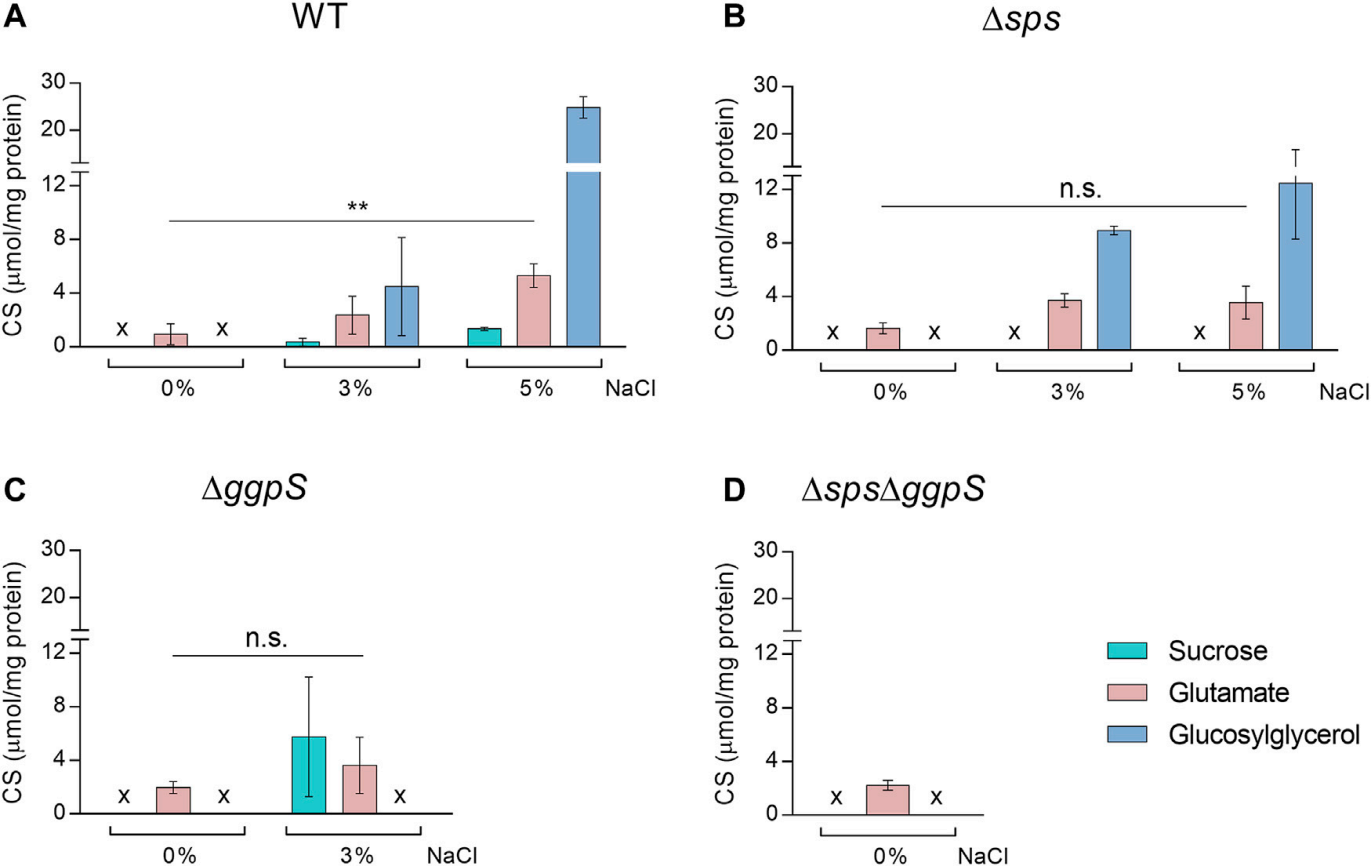
Effect of NaCl on the synthesis of native compatible solutes sucrose, glutamate and glucosylglycerol by *Synechocystis* wild-type (WT) **(A)**, and the ∆*sps* **(B)**, ∆*ggpS* **(C),** and ∆*sps*∆*ggpS* mutants **(D)**. Cultures were grown in BG11 or BG11 supplemented with 3% or 5% (wt/vol) NaCl, at 30°C with orbital shaking (150 rpm) under a 12 h light (25 μE/m^2^/s)/12 h dark regimen, and cells were harvested 4 days after inoculation (initial OD_730_ ≈ 0.5). Compatible solutes were quantified by H-NMR and the results were normalized per mg of protein. x—not detected. Error bars correspond to standard deviations from at least three biological replicates. Statistical analysis was performed using two-way ANOVA. Statistically significant differences are identified: ** (*p* ≤ 0.01) and n.s. (not significant).

The CS content was quantified in *Synechocystis* WT, ∆*sps*, ∆*ggpS*, and ∆*sps*∆*ggpS* mutants grown in BG11 or BG11 supplemented with NaCl (Figure 2), under salinity conditions in which each strain could sustain growth (Figure 1). In the WT, CS accumulation increased significantly in a salinity-dependent manner and GG was accumulated in higher amounts followed by glutamate and sucrose (Figure 2A). As expected, the two main compatible solutes sucrose and GG could only be detected in the presence of NaCl. The amino acid glutamate was detected in all backgrounds and conditions, and increased more than 1.8-fold in the presence of salinity (significant difference *p* ≤ 0.01 for WT in 0 and 5% NaCl). In the ∆*sps*, ∆*ggpS*, and ∆*sps*∆*ggpS* mutants the absence of sucrose and/or GG production was confirmed (Figures 2B–D). For the ∆*sps*, a salinity-dependent accumulation of GG was also detected (Figure 2B), however, the GG concentration was 50% lower compared with the WT, under 5% NaCl. In contrast, the ∆*ggpS* mutant accumulated 17-fold more sucrose than the WT, under 3% NaCl (Figure 2C). All the proton NMR spectra are depicted in Additional file 1: Supplementary Figure S3.

### Effect of NaCl on Total Carbohydrates, Glycogen, Capsular, and Released Polysaccharides

**FIGURE 3:**
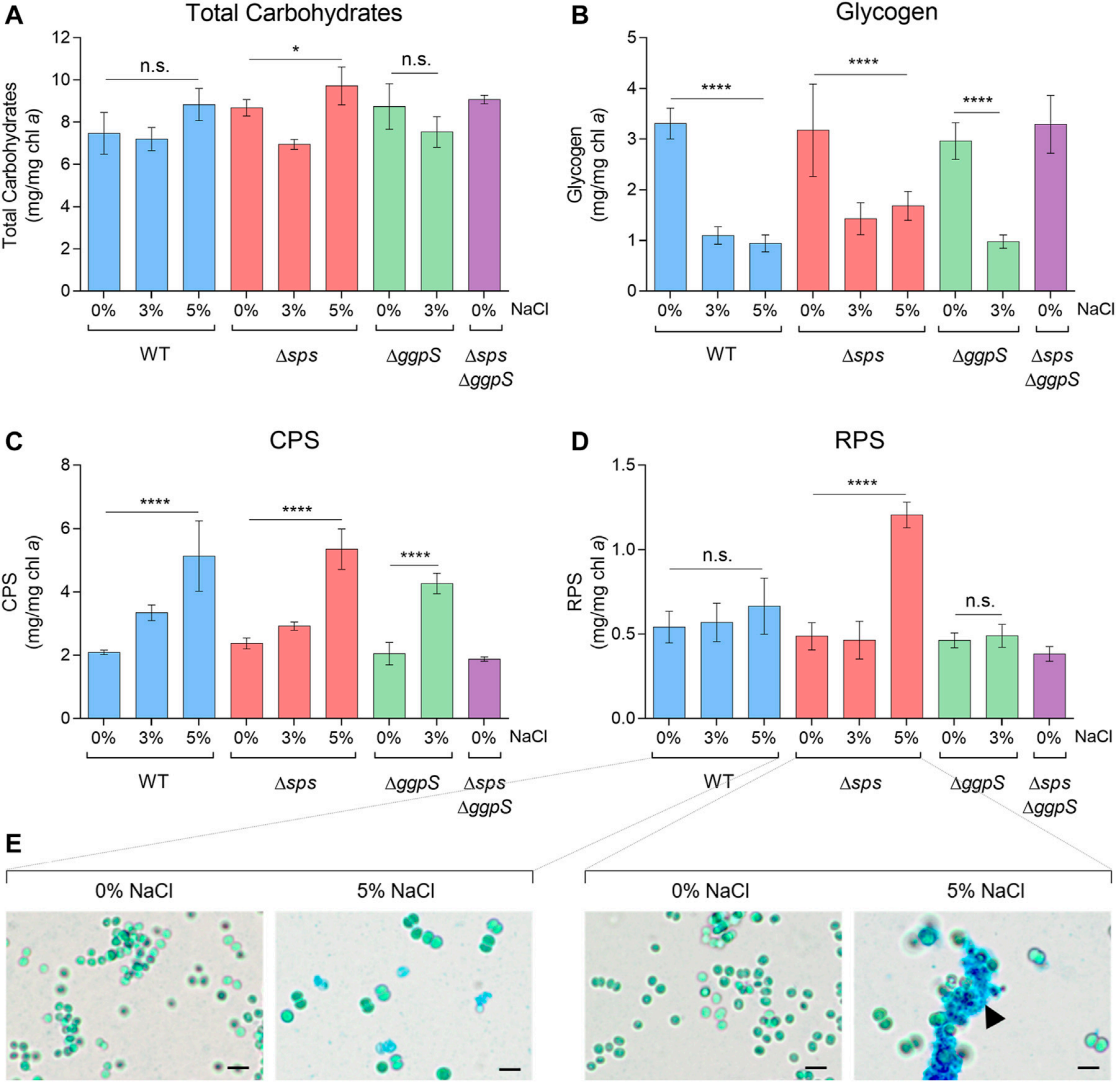
Effect of NaCl on total carbohydrates **(A)**, glycogen **(B)**, CPS—capsular polysaccharides **(C)**, and RPS—released polysaccharides **(D)** produced by *Synechocystis* wild-type (WT) and the ∆*sps*, ∆*ggpS*, and ∆*sps*∆*ggpS* mutants. Cells were grown in BG11 or BG11 supplemented with 3% or 5% (wt/vol) NaCl, at 30°C with orbital shaking (150 rpm) under a 12 h light (25 μE/m^2^/s)/12 h dark regimen for 16 days. Results are expressed as milligrams per milligram of chlorophyll *a* (chl *a*). Error bars correspond to standard deviations from at least three biological replicates, with technical triplicates. Statistical analysis was performed using one-way ANOVA. Statistically significant differences are identified: **** (*p* ≤ 0.0001), * (*p* ≤ 0.05), and n.s. (not significant). Light micrographs **(E)** of *Synechocystis* WT and ∆*sps* cultures grown in BG11 or BG11 supplemented with 5% NaCl and stained with Alcian Blue; the black arrowhead highlights RPS production by the ∆*sps* mutant under 5% NaCl. Scale bars: 5 µm.

In addition to the CS pools, the total carbohydrate content was analyzed in *Synechocystis* WT and the CS deficient mutants (Figure 3A). Generally, the presence of salinity (3 or 5% NaCl) had no significant impact on the production of total carbohydrates, except for the ∆*sps* mutant that showed some fluctuation when exposed to different salinities (Figure 3A; *p* ≤ 0.05). To further clarify the carbon distribution in response to salinity, the amount of glycogen as well as the production of extracellular polymeric substances, CPS and RPS, were also determined (Figures 3B–D). The presence of NaCl led to a significant decrease in the amount of glycogen in the WT, ∆*sps*, and ∆*ggpS*, with reductions of more than 56%, independent of the salinity concentration and the deletion of one of the CS pathways (Figure 3B). In terms of CPS, the opposite effect was observed with a 2.4-fold increase in CPS for the WT and the ∆*sps* at 5%, and a 2.1-fold increase for ∆*ggpS* at 3% NaCl, compared with 0% NaCl (Figure 3C). The amount of RPS produced by WT or ∆*ggpS* did not change significantly whereas for the ∆*sps* a 2.5-fold increase was registered under 5% NaCl (Figure 3D). Staining the WT and ∆*sps* cultures with Alcian Blue confirmed similar RPS production for the WT under 0 and 5% NaCl, while for the ∆*sps* the accumulation of RPS in 5% NaCl is evident, leading to the formation of cell aggregates (Figure 3E; black arrowhead).

### *In silico* Prediction of Production Rates for Native and Heterologous CS Using *Synechocystis* Wild-Type

**FIGURE 4:**
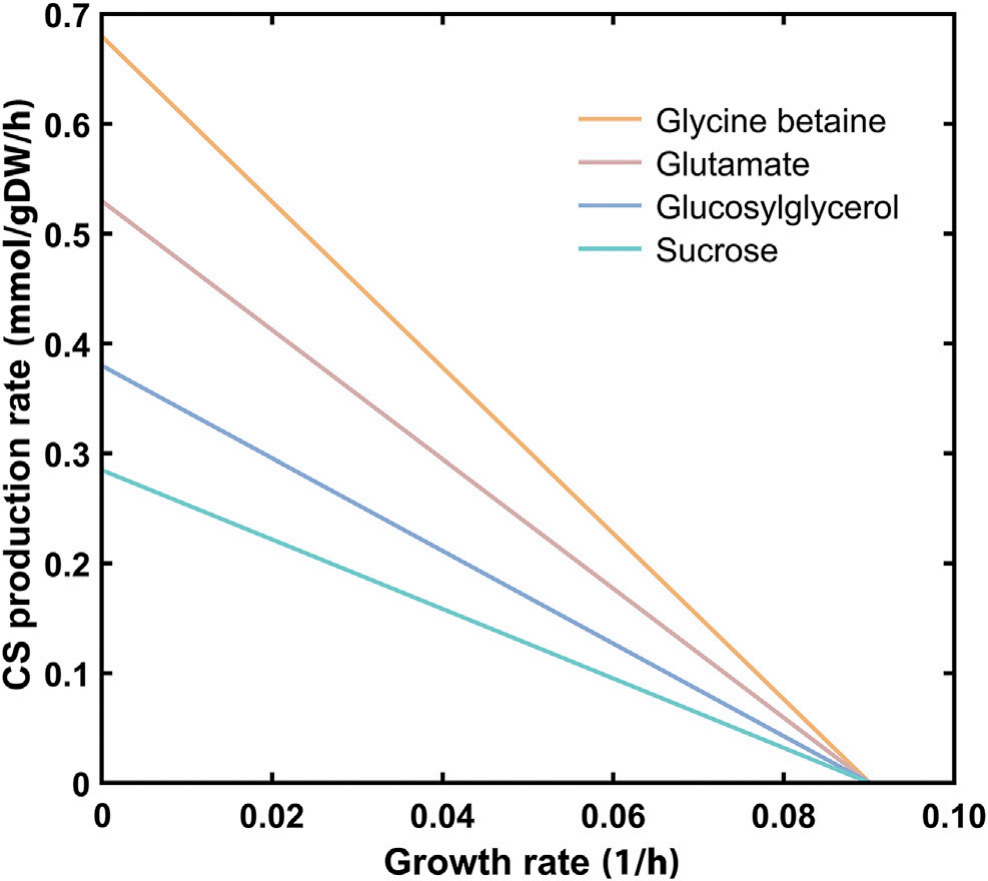
Theoretical productivity of the heterologous compatible solute glycine betaine and the native ones (glutamate, glucosylglycerol, and sucrose), as predicted by the updated version of the genome-scale metabolic model *i*Syn811. The lines represent the compatible solute production rate as a function of *Synechocystis* wild-type growth under autotrophic conditions.

The genome-scale metabolic model of *Synechocystis*—*i*Syn811 (Montagud et al., 2011)—was updated to include all the information required for calculating CS production rates. The manual curation process started with the addition of the metabolic precursors and the reactions required for CS synthesis. The nomenclature was also corrected and standardized, and the reversibility of some reactions was changed (for more details see the *Materials* section). After the curation of the metabolic model was completed, the COBRA (“The COnstraint-Based Reconstruction and Analysis”) Toolbox v3.0 (Heirendt et al., 2019), was used to simulate the compatible solute production rate as a function of *Synechocystis* wild-type growth under autotrophic conditions (Figure 4). The results show a linear tradeoff between the cell’s resources toward growth or the production of the different CS. Regarding the production of the native CS, GG, and sucrose impose a higher metabolic burden showing lower *in silico* production rates (0.378 and 0.283 mmol/gDW/h, respectively), compared with glutamate (0.567 mmol/gDW/h). The simulation of the production of heterologous CS glycine betaine (GB) showed the best compromise between growth and production compared with the three native CS, with the highest predicted maximum production rate of 0.680 mmol/gDW/h. In addition to GB, the production rates of other heterologous CS, such as ectoine and mannosylglycerate were also simulated, revealing that the maximum production rate predicted for these two solutes is lower than the obtained for GB (Additional file 1: Supplementary Figure S4). Hence, GB was chosen as the heterologous CS to be produced using the *Synechocystis* chassis developed (CS-deficient mutants).

### Design and Assembly of the Synthetic Device for the Production of Glycine Betaine

**FIGURE 5:**
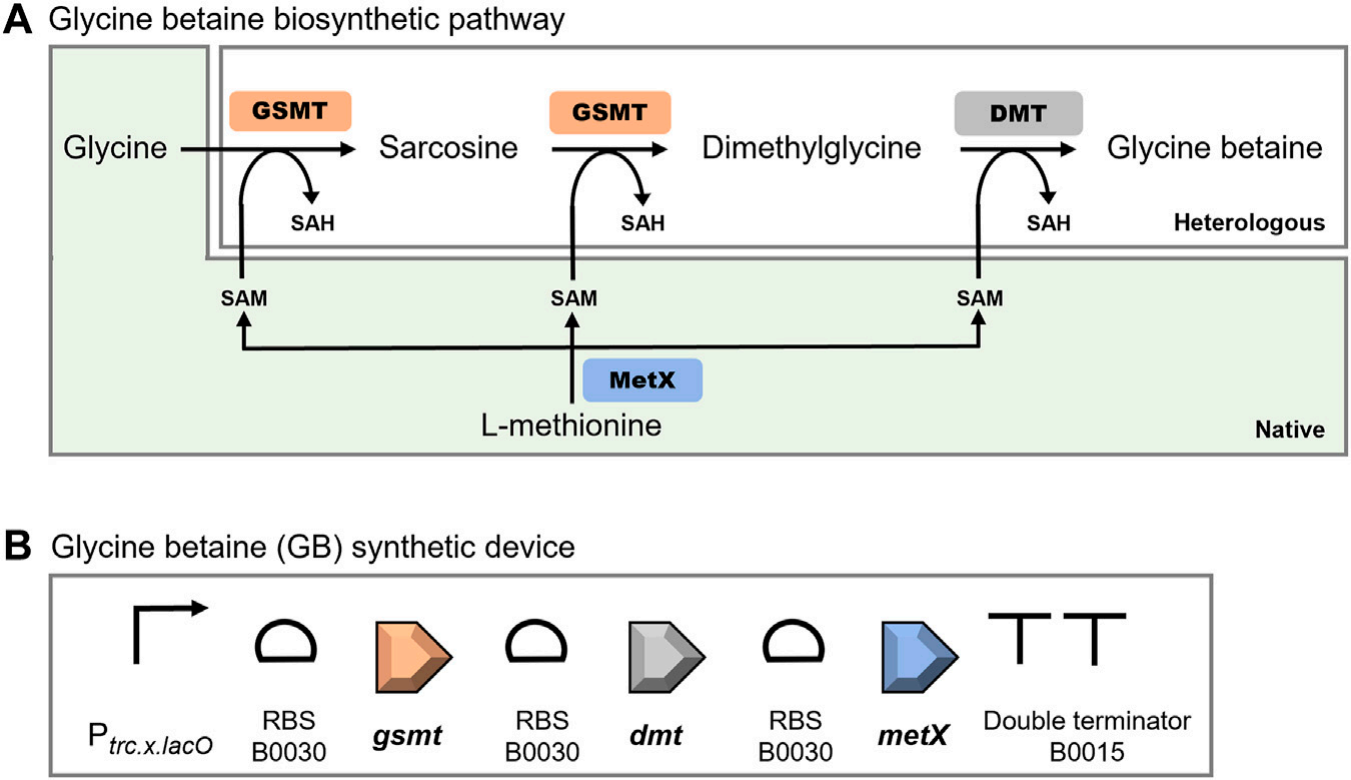
Glycine betaine biosynthetic pathway **(A)** and schematic representation of the glycine betaine (GB) synthetic device for the production of this heterologous compatible solute in *Synechocystis* **(B)**. The glycine betaine biosynthetic pathway comprises the GSMT (glycine-sarcosine-*N*-methyltransferase) and DMT (dimethylglycine-*N*-methyltransferase) from *Aphanothece halophytica* (heterologous), and MetX (*S*-adenosyl-methionine synthase) from *Synechocystis* (native). SAM—*S*-adenosyl-methionine; SAH—*S*-adenosyl-homocysteine. The GB device includes the promoter P_*trc.x.lacO*_, the ribosomal binding site (RBS) B0030, the open reading frames *gsmt*, *dmt*, and *metX* codon optimized for *Synechocystis*, and the double terminator B0015.

Envisaging the heterologous production of GB, a synthetic device was designed based on the metabolic pathway described for the halophilic cyanobacterium *Aphanothece halophytica* (Figure 5A) (Nyyssola et al., 2000; Waditee et al., 2003). This device comprises two Open Reading Frames (ORFs) encoding the enzymes involved in the three-step methylation of glycine to glycine betaine: glycine-sarcosine-*N*-methyltransferase (*gsmt*), and the dimethylglycine-*N*-methyltransferase (*dmt*). In these reactions, *S*-adenosylmethionine (SAM) is the source of methyl groups for the synthesis of GB, and it can be synthesized from L-methionine by the *S*-adenosyl-methionine synthase (MetX) (Figure 5A). To prevent the shortage of the SAM precursor, the ORF encoding *Synechocystis’* native MetX (*metX*, *sll0927*) was also included in the device. The sequences of the three ORFs (*gsmt*, *dmt*, and *metX*) were codon-optimized and restriction sites incompatible with the BioBrick standard RFC [10] were eliminated. Subsequently, the ribosome binding site (RBS) BBa_B0030 and double stop codons (TAATAA) were included before and after each ORF, respectively. A double terminator (BBa_B0015) was also included at the end of the synthetic construction (Ahbet). Additionally, the designed DNA sequence was flanked by the prefix and suffix of the BioBrick RFC [10] standard (Canton et al., 2008), enabling the use of the standard assembly method to include the regulatory element (promoter). After DNA synthesis, the Ahbet construction was cloned downstream of the promoter P_*trc.x.lacO*_, originating the P_*trc.x.lacO*_::Ahbet synthetic device (hereafter GB device) (Figure 5B). The P_*trc.x.lacO*_ is a constitutive promoter in *Synechocystis*, previously characterized by our group and is 41-fold stronger than the reference cyanobacterial promoter P_*rnpB*_ (Ferreira et al., 2018).

### Effect of the Implementation of the GB Device Into the *Synechocystis* Chassis

**FIGURE 6:**
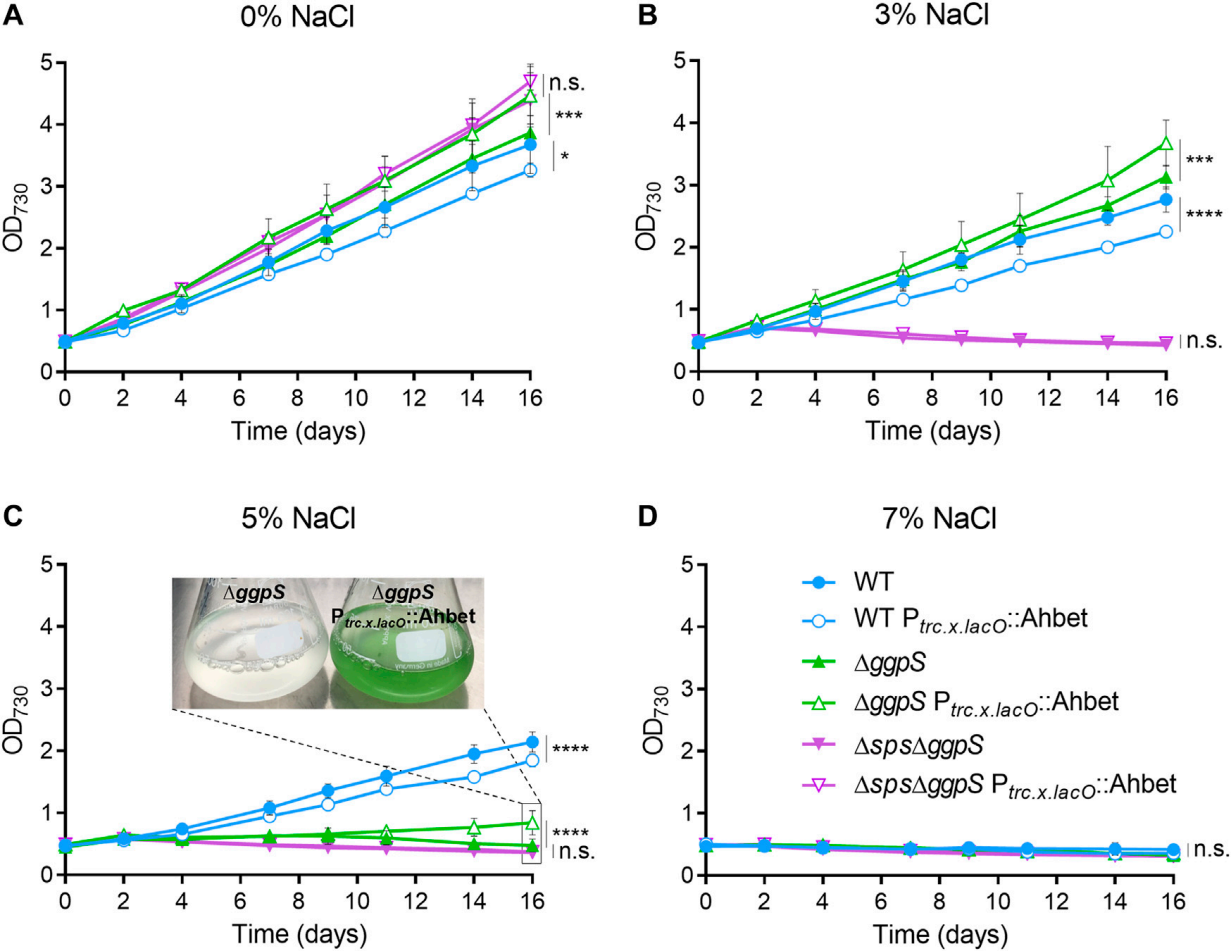
Growth of *Synechocystis* wild-type (WT), ∆*ggpS*, and ∆*sps*∆*ggpS* without or with the GB device (P_*trc.x.lacO*_::Ahbet). Cultures were grown in BG11 **(A)** or BG11 supplemented with 3% **(B)**, 5% **(C)**, or 7% **(D)** (wt/vol) NaCl, at 30°C with orbital shaking (150 rpm) under a 12 h light (25 μE/m^2^/s)/12 h dark regimen. Growth was monitored by measuring the optical density at 730 nm (OD_730_). Error bars correspond to standard deviations from at least three biological replicates with technical duplicates. Statistical analysis was performed using two-way ANOVA. Statistically significant differences are identified: **** (*p* ≤ 0.0001), *** (*p* ≤ 0.001), * (*p* ≤ 0.05), and n.s. (not significant). In Figure 6C, the liquid cultures of *Synechocystis* ∆*ggpS* **(left)** and ∆*ggpS* P_*trc.x.lacO*_::Ahbet **(right)**, in BG11 supplemented with 5% NaCl after 16 days of cultivation, are shown.

The GB synthetic device was implemented into the *Synechocystis* WT and the CS deficient ∆*ggpS* and ∆*sps*∆*ggpS* chassis described above, using the replicative plasmid pSEVA351. The device was not introduced into ∆*sps* since the characterization showed that this mutant is similar to the WT in terms of growth, total carbohydrates, glycogen content and CPS (Figure 1, Figures 3A–C, respectively). The presence of the GB device in the cells was confirmed by PCR (Additional file 1: Supplementary Figure S5), and the growth and chl *a* content of the transformants were monitored in absence/presence of salinity and compared with the respective backgrounds (Figure 6; Additional file 1: Supplementary Figure S6). As shown in Figure 6, the introduction of the synthetic device had distinct effects depending on the genetic background. The implementation of GB device into ∆*ggpS* led to a significant improvement of growth (16%) in BG11 and BG11 supplemented with 3% NaCl (Figures 6A,B; green lines), and supported its survival under 5% NaCl (Figure 6C; green lines). After 16 days of cultivation under 5% NaCl, the batch culture of ∆*ggpS* showed clear signs of chlorosis/necrosis while the culture of the ∆*ggpS* mutant harboring the GB device remained green (Figure 6C; insert). In agreement, the chl *a* content was 0.8 µg/ml and 1.8 µg/ml, respectively (Additional file 1: Supplementary Figure S6; green lines). Notably, this survival phenotype was observed for at least 25 days (data not shown). In the WT background the presence of the device had a detrimental effect on growth (∼15% decrease) in all conditions tested (Figure 6; blue lines). The growth of the double mutant ∆*sps*∆*ggpS* in the absence of NaCl was not affected by the introduction of the synthetic device (Figure 6A; purple lines). Moreover, the mutant harboring the GB device was unable to survive under saline conditions, similarly to what happened to ∆*sps*∆*ggpS* background (Figures 6B–D; purple lines).

### Analysis of Transcript Levels in *Synechocystis* Strains Harboring the GB Device

**FIGURE 7:**
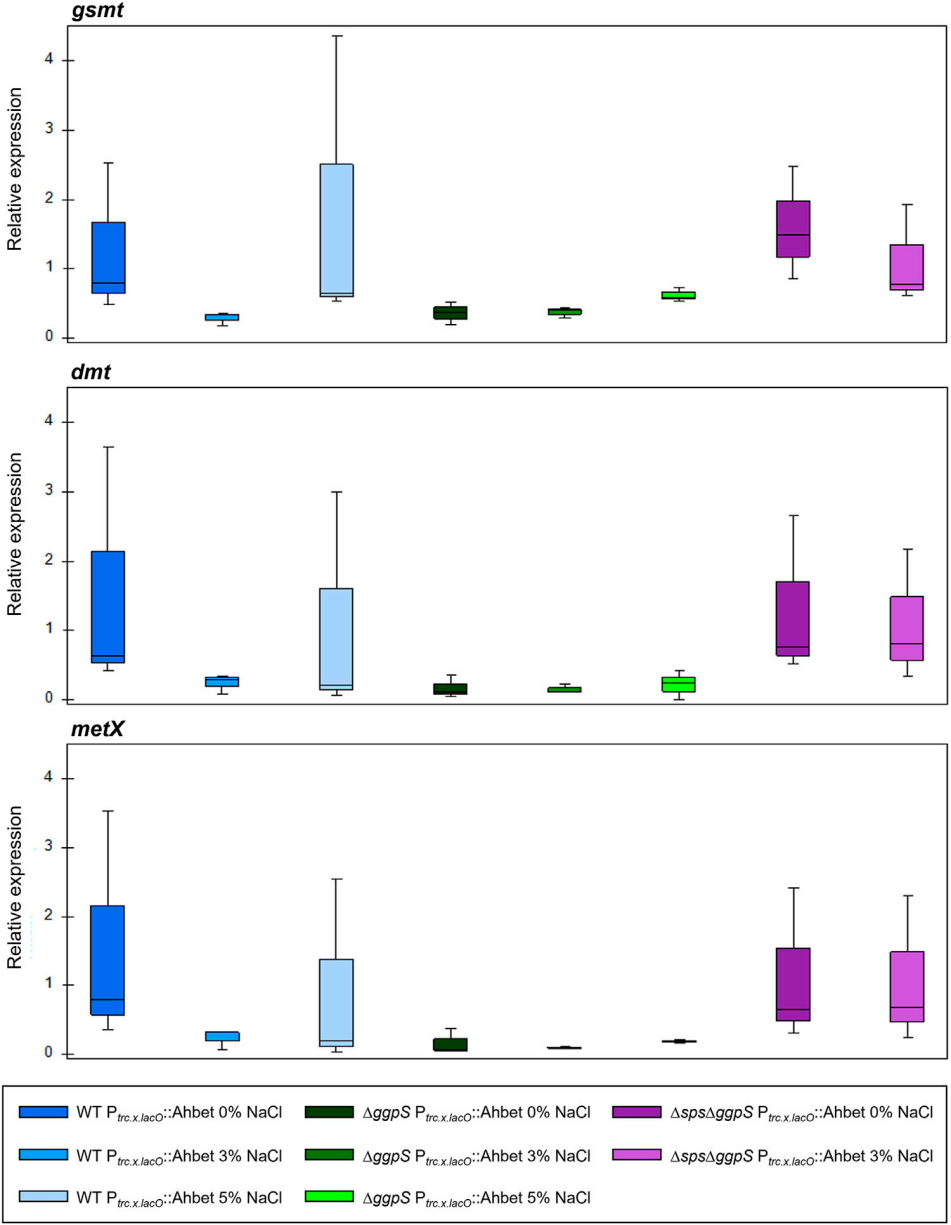
RT-qPCR analysis of *gsmt*, *dmt*, and *metX* relative expression in *Synechocystis* strains (WT, ∆*ggpS*, and ∆*sps*∆*ggpS*) harboring the GB device. RNA was extracted from cells grown in BG11 or BG11 supplemented with 3 and 5% (wt/vol) NaCl, at 30°C with orbital shaking (150 rpm) under a 12 h light (25 μE/m^2^/s)/12 h dark regimen. The box-whisker plots represent the expression of the target genes relative to WT P_*trc.x.LacO*_::Ahbet in absence of salt (0% NaCl). Data were obtained from three biological replicates and three technical replicates, and the whiskers represent the minimum and maximum non-outlier values in the dataset. One-way ANOVA was performed no significant differences could be detected.

The next step in the characterization of the different *Synechocystis* strains harboring the GB device was the evaluation of the transcript levels of the three ORFs comprised in the device (*gsmt*, *dmt*, and *metX*) by RT-qPCR (for more details see the *Materials* section). As shown in Figure 7, the transcripts of the three genes (*gsmt*, *dmt*, and *metX*) were detected in all samples and the relative expression was reasonably stable independently of the background strain. Additionally, the relative expression remained similar under salinity conditions and, even though some variation could be detected, it was not statistically significant (Additional file 1: Supplementary Table S2).

### Quantification of Native and Heterologous CS in *Synechocystis* Chassis Harboring the GB Device

**FIGURE 8:**
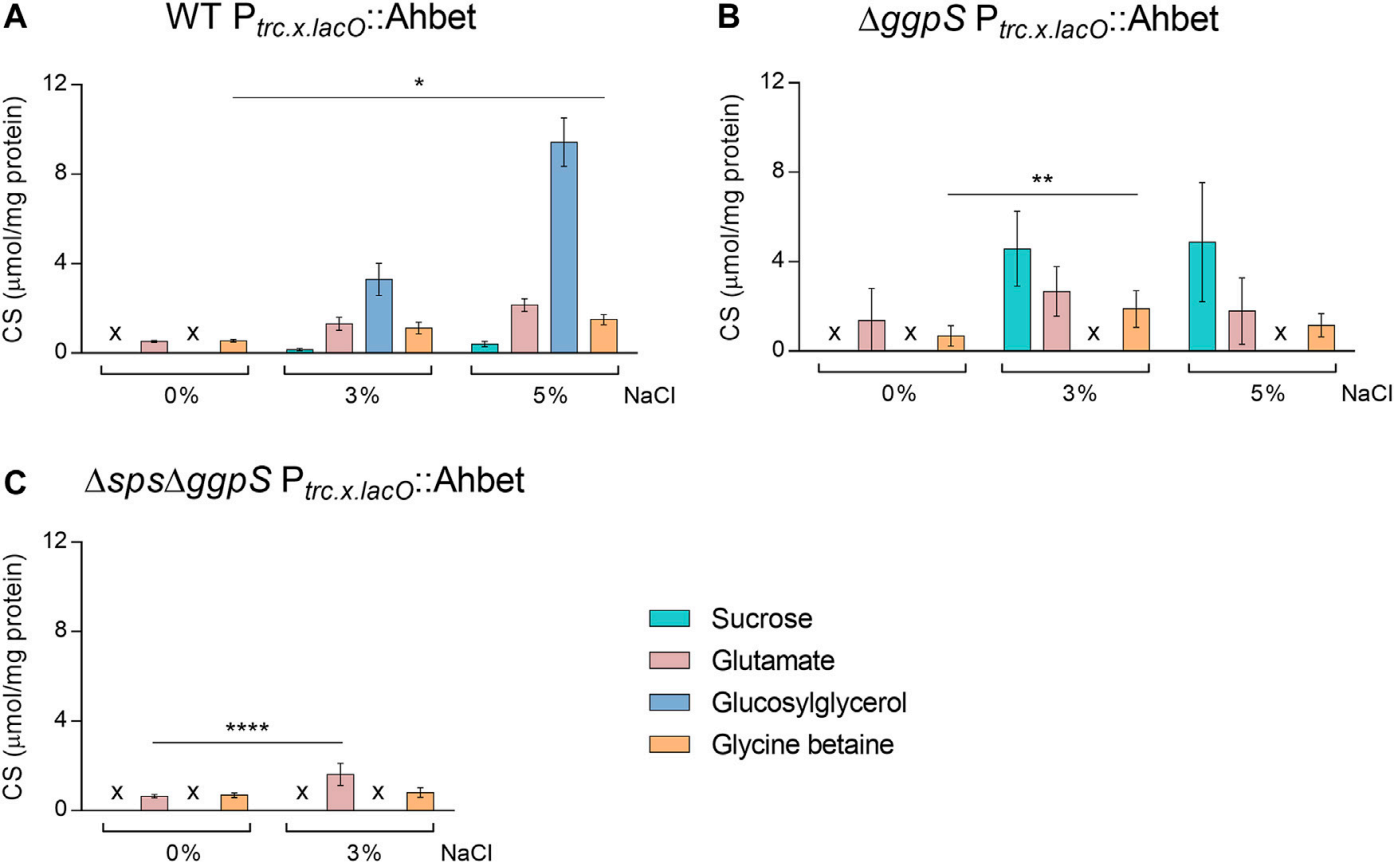
Production of glycine betaine (GB) and native compatible solutes in different *Synechocystis* strains harboring the GB device: WT (wild-type) P_*trc.x.lacO*_::Ahbet **(A)**, ∆*ggpS* P_*trc.x.lacO*_::Ahbet **(B)**, and ∆*sps*∆*ggpS* P_*trc.x.lacO*_::Ahbet **(C)**. Cultures were grown in BG11 or BG11 supplemented with 3% or 5% (wt/vol) NaCl, at 30°C with orbital shaking (150 rpm) under a 12 h light (25 μE/m^2^/s)/12 h dark regimen; and cells were harvested 4 days after inoculation (initial OD_730_ ≈ 0.5). Compatible solutes were quantified by H-NMR and the results were normalized per mg of protein. x—not detected. Error bars correspond to standard deviations from three biological replicates. Statistical analysis was performed using two-way ANOVA. Statistically significant differences are identified: **** (*p* ≤ 0.0001), ** (*p* ≤ 0.01), and * (*p* ≤ 0.05).

The CS pool of the different *Synechocystis* strains (WT, ∆*ggpS*, and ∆*sps*∆*ggpS*) harboring the GB device was analyzed in absence/presence of NaCl after 4 days of cultivation (Figure 8). The results obtained confirmed that the implementation of the pathway for the synthesis of heterologous CS was successful, since glycine betaine could be detected in all strains and conditions analyzed. Under 0 and 3% NaCl, the presence of the GB device in the WT background did not significantly influence the synthesis of native CS and heterologous production of GB is not significantly influenced by salinity. However, under 5% NaCl, there was an impact on the synthesis of glutamate and GG that decreased by 59 and 62%, respectively, and the synthesis of GB increased 2.7-fold compared with 0% NaCl (Figures 2A, 8A). Similarly, the implementation of the device in the ∆*ggpS* did not affect the production of the native CS under 0 and 3% NaCl compared with ∆*ggpS* chassis (Figures 2C, 8B). However, the introduction of the device into the ∆*ggpS* background allowed this strain to survive under 5% NaCl and, therefore the quantification of CS was also performed. The results obtained showed that in ∆*ggpS* P_*trc.x.lacO*_::Ahbet, besides glycine betaine production, the levels of sucrose and glutamate were similar to the ones observed for 3% NaCl (Figure 8B). An analysis of the ∆*ggpS* harboring the GB device after 16 days of cultivation suggested that the production of all CS is maintained for at least 2 weeks of cultivation (Additional file 1: Supplementary Figure S7). For the double mutant, the presence of the device led to a significant decrease (71%) of glutamate in the absence of salinity (Figures 2D, 8C). In contrast, under 3% NaCl, there was a 2.5-fold increase in glutamate (*p* ≤ 0.0001) compared with 0% NaCl, while the glycine betaine content remained similar (Figure 8C). All the proton NMR spectra are depicted in Additional file 1: Supplementary Figure S8. Furthermore, the CS quantification was also performed for the extracellular medium, and the results showed that none of the native CS could be detected, while GB was detected in negligible amounts in all strains harboring the device and conditions tested (Additional file 1: Supplementary Table S3).

## Discussion

The sustainable production of compatible solutes (CS) is essential for pharmaceutical and cosmetic industries. The current microbiological processes have a significant negative impact on the environment, which could be mitigated by the use of photoautotrophic chassis such as cyanobacteria. For the synthesis of heterologous CS in *Synechocystis*, the construction of customized chassis is required and our strategy was to eliminate competing or redundant pathways. Therefore, in this work we have generated three *Synechocystis* mutants deficient in the production of native compatible solutes (namely, sucrose, or/and glucosylglycerol). These strains—Δ*sps*, Δ*ggpS*, and Δ*sps*Δ*ggpS*—were characterized under different salinity concentrations, expanding the knowledge that will allow further optimization of the chassis for the increased production of heterologous CS, such as glycine betaine (GB). In this context, an updated version of the genome-scale metabolic model of *Synechocystis*—*i*Syn811 (Montagud et al., 2011)—was used to predict the production rates for native and heterologous CS using *Synechocystis* wild-type. The simulations show a linear tradeoff between deviating resources toward cellular growth or toward the production of the solutes. Since energy and carbon uptake are limited, any extra need of ATP or carbon molecules for compatible solute synthesis will impair cell growth. Whether carbon or light uptake is limiting the synthesis of each CS is difficult to predict, since alternative routes with different energetic efficiencies can be simultaneously active under different growth conditions. From the CS evaluated, the predictions indicate that the synthesis of native sucrose, and glucosylglycerol (GG) has a higher impact on cell growth than glutamate or the heterologous solute glycine betaine (GB) (Figure 4). The production of sucrose and GG require glucose that drains more cell resources than the reported for the synthesis of an amino acid (Kaleta et al., 2013), such as glutamate or glycine (the latter required for GB production). The results also suggest that the production of GB has a smaller restraining effect on growth than glutamate or other heterologous CS, like ectoine and mannosylglycerate (Additional file 1: Supplementary Figure S4).

In parallel, the evaluation of the CS levels of the wild-type and the mutants (Δ*sps*, Δ*ggpS*, and Δ*sps*Δ*ggpS*) confirmed the salt-induced accumulation of sucrose and GG, which is well documented in the literature [for reviews see e.g*.*, Klähn and Hagemann (2011); Hagemann (2013); Kirsch et al. (2019)]. In contrast, glutamate could be detected in the absence and presence of NaCl (Figure 2). These results are in agreement with the reported accumulation of this amino acid in *Synechocystis* grown in artificial seawater medium (ASW; 340 mM NaCl), and in BG11 supplemented with 12 mM KCl (Iijima et al., 2015; Iijima et al., 2020).

Previous works have also reported a tradeoff between the pools of different compatible solutes and other carbon sinks, such as glycogen or extracellular polymeric substances (EPS) in cyanobacteria (Du et al., 2013; Baran et al., 2017; Kirsch et al., 2017). In our work, the total carbohydrate content of *Synechocystis* WT and CS-deficient mutants remained unchanged when cells were exposed to NaCl, whereas a significant decrease in glycogen was observed (Figure 3). Concomitantly, the accumulation of capsular polysaccharides (CPS) was observed in a salinity-dependent manner and for the strains tested. In line with these observations is the increase in the levels of proteins involved in glycogen degradation, reported when *Synechocystis* cells were grown in ASW medium (Iijima et al., 2015). The protective role of EPS against salt stress was also demonstrated in a *Synechocystis* ∆*sll1581*∆*slr1875* double mutant, showing that a decrease in CPS content increases NaCl sensitivity (Jittawuttipoka et al., 2013). Altogether, these results strongly suggest that under saline conditions, *Synechocystis* breaks down glycogen and redirects carbon fluxes toward the production of CS and extracellular polysaccharides, promoting cell homeostasis and contributing to cell protection.

From the three *Synechocystis* CS-deficient mutants generated in this work, the Δ*sps* was the only one able to grow in 5% NaCl. We also observed that this mutant’s growth gets impaired over time, suggesting that the presence of sucrose is of additional importance for long-term cultivation. Accordingly, a previous work showed that Δ*sps* cells in stationary phase were unable to survive a salt shock, which was not observed for cells in exponential phase; this effect could be prevented by sucrose supplementation (Desplats et al., 2005). Moreover, we show that the absence of sucrose leads to a severe reduction in the accumulation of GG whereas the released polysaccharides (RPS) increase significantly (1.8-fold), implying that extracellular polysaccharides production is crucial for the survival of the Δ*sps* mutant under 5% NaCl. These results also suggest that sucrose role might go beyond osmoprotection, being involved in the regulation of metabolic pathways, e.g., triggering signaling cascades, as it was previously hypothesized by Desplats et al. (2005). For the ∆*ggpS* mutant an increased sucrose level was detected under 3% NaCl, showing that this sugar can sustain *Synechocystis’* survival under sea salt conditions for at least 16 days. Previously (Miao et al., 2003), generated a *Synechocystis* ∆*agp* mutant unable to synthesize ADP-glucose (a precursor required for GG synthesis) that was also shown to accumulate high levels of sucrose and could survive upon a salt shock of 900 mM (5.2% NaCl). Notably, in the latter work and here, the mutant’s sucrose levels were similar to GG accumulated in the WT cultivated under the same conditions. Taken together, these studies imply that GG and sucrose can have comparable osmoprotectant capacity when accumulated in similar levels. Additionally, the ∆*sps*∆*ggpS* mutant was unable to survive in any salt concentration tested and glutamate was the only CS that could be detected. Thus, this amino acid seems to have a minor contribution to the salt acclimation process in *Synechocystis*, similar to what was reported for the halophilic bacterium *Salinibacter ruber* (Oren et al., 2002).

Considering the metabolic model simulation, a synthetic device for the production of glycine betaine (GB) was designed and implemented into the *Synechocystis* wild-type and our customized chassis (CS-deficient mutants). Besides the ORFs required for GB production (*gsmt* and *dmt*) and *metX* (to prevent SAM shortage), this device comprises well-characterized regulatory elements: the synthetic promoter P_*trc.x.lacO*_ (Ferreira et al., 2018), the RBS BBa_B0030, and the double terminator BBa_B0015. This design ensured the stable constitutive transcription observed for the GB device ORFs, regardless of the chassis or salinity conditions (Figure 7), reinforcing that the use of orthogonal regulatory components is crucial to ensure the proper insulation of synthetic devices from the regulatory network of the chassis (Costello and Badran, 2021). Unlike transcription, the synthesis of the solute was not independent of cultivation conditions, and GB levels increased with salinity. In agreement, higher levels of glycine have been reported for *Synechocystis* cells grown in ASW medium compared with those grown in BG11 (Iijima et al., 2015). Since this amino acid is a precursor of GB, the high levels of glycine under salinity conditions most probably favor the synthesis of GB. In addition, glycogen degradation and carbon fluxes redirection toward the production of CS could also explain the increased amount of GB produced in the presence of NaCl.

The implementation of the GB device into *Synechocystis* wild-type led to a small decrease in growth in all conditions tested (Figure 6). As predicted by the metabolic flux model, the device may drain the cell’s resources imposing a metabolic burden, causing growth impairment. This can be explained by the redirection of part of the photosynthetically fixed carbon to the synthesis of CS, which is no longer available for biomass formation, similarly to what was reported for the production of mannitol (Wu et al., 2020). In contrast to what was observed for the WT, the introduction of the GB device into the Δ*ggpS* mutant resulted in an increased salt tolerance with the concomitant growth improvement, enabling its survival under 5% NaCl. This phenotype was maintained under long-term cultivation periods up to 25 days (data not shown), suggesting that GB can compensate for the absence of GG. Conversely, the implementation of the GB device in the ∆*sps*∆*ggpS* mutant did not improve its performance under salinity conditions. However, it remains unclear if this outcome is due to: 1) insufficient production of glycine betaine to allow cell survival or 2) the absence of both native compatible solutes (sucrose and GG).

In terms of production, the highest GB amount was obtained for the Δ*ggpS* cultivated in BG11 supplemented with 3% NaCl for 4 days (1.89 μmol GB/mg protein, corresponding to 64.29 μmol/gDW, and a volumetric productivity of 13.67 µg/L/h) (Figure 8B). Unexpectedly, the production of GB was not higher at 5% NaCl, which may be due to the limited capacity of the cells to survive in such conditions. Extending the cultivation period up to 16 days does not seem to affect the synthesis of GB in any condition tested (Supplementary Figure S7), suggesting that the process is stable. In addition, the negligible GB amounts detected in the extracellular medium show that this CS can be exported. Since product secretion facilitates recovery and reduces costs, this aspect should be addressed in the establishment of a GB-cell factory.

The production of GB using native organisms and heterologous hosts (with the synthesis of the solute mainly based on the metabolic pathway described for *A. halophytica*)*,* has been previously reported (Table 2). However, a direct comparison is difficult since different normalization methods were used, and the cultivation conditions/time periods need also to be taken into consideration. Generally, the use of native GB producers such as the hypersaline cyanobacterium *A. halophytica* can render high amounts of the solute. This entails major disadvantages related to the high salt concentrations required, such as the reduced durability of the bioreactors, long processes, and detrimental impact on the environment. In contrast, with heterologous hosts the salinity concentrations used are at least 1/3 of those employed for *A. halophytica* (Table 2—heterologous production)*.* Considering the photoautotrophic organisms, the amounts obtained using plants are low and require rather long cultivation periods. The most promising results were obtained using the filamentous cyanobacterium *Anabaena doliolum* that produced 12.92 µmol GB/gDW after 10 days of cultivation (Singh et al., 2013). Using our *Synechocystis* Δ*ggpS* chassis, we report a production level ∼5-fold higher than that of *A. doliolum* in just 4 days of cultivation (64.29 µmol GB/gDW). Regarding the heterotrophic chassis, the GB amounts obtained using different *Escherichia coli* strains were only up to 1.25-fold higher than with *Synechocystis* Δ*ggpS*. Cultivation times are significantly reduced for heterotrophic bacteria, but the use of photoautotrophic chassis enables CO_2_ fixation promoting bio-mitigation and surpassing the need to supply a carbon source. Additionally, the highest GB production by our *Synechocystis* Δ*ggpS* chassis was achieved under 510 mM NaCl, opening up the possibility of large-scale cultivation with seawater (salinity range 3.1–3.8%). This does not seem as viable with *E. coli* since increasing the salt concentration to 500 mM has a substantial detrimental impact on the GB production (Waditee-Sirisattha et al., 2012).

**TABLE 2:** Native and heterologous production of glycine betaine *via* the three-step glycine methylation pathway.

Native production				
Strain	Salinity (mM)	Production capacity	Cultivation time	Reference
*Aphanothece halophytica*	2,000	0.06 µmol/mg protein	1 h	Ishitani et al. (1993)
*Aphanothece halophytica*	2,000	∼0.4 µmol/10^7^ cells	7 days	Incharoensakdi and Waditee (2000)
*Aphanothece halophytica*	1,500	∼40,000 µmol/gFW	10 days	Waditee et al. (2007)
*Aphanothece halophytica*	2,000	20.1 µmol/gFW	15 days	Waditee-Sirisattha et al. (2015)

Heterologous production					
Production strain	Native strain	Salinity (mM)	Production capacity	Cultivation time	Reference
*Arabidopsis thaliana*	*Aphanothece halophytica*	100	∼2 µmol/gFW	15 days	Waditee et al. (2005)
*Nicotiana tabacum*	*Aphanothece halophytica*	0	0.4 µmol/gFW	28 days	He et al. (2011)
*Synechococcus* PCC 7942	*Aphanothece halophytica*	500	∼1.5 µmol/gFW	NA	Waditee et al. (2005)
*Anabaena* sp. PCC 7120	*Aphanothece halophytica*	140	0.04 µmol/gFW	7 days	Waditee-Sirisattha et al. (2012)
*Anabaena doliolum*	*Aphanothece halophytica*	500	12.92 µmol/gDW	10 days	Singh et al. (2013)
*Synechocystis* sp. PCC 6803	*Aphanothece halophytica*	510	64.29 µmol/gDW	4 days	This work
*Escherichia coli* XL1-Blue	*Ectothiorhodospira halochloris*	300	78 µmol/gDW	NA	Nyyssola et al. (2000)
*Escherichia coli* BL21	*Aphanothece halophytica*	300	∼23 µmol/gDW	3 h	Waditee et al. (2003)
*Escherichia coli* BL21	*Aphanothece halophytica*	300	∼2,000 µmol/mg protein	3 h	Waditee et al. (2007)
*Escherichia coli* BL21	*Aphanothece halophytica*	300	∼80 µmol/L	2 h	He et al. (2011)
*Escherichia coli* DH5α	*Aphanothece halophytica*	500	6 µmol/gDW	24 h	Waditee-Sirisattha et al. (2012)
*Escherichia coli* DH5α	*Aphanothece halophytica*	0	80.62 µmol/gDW	ON	Singh et al. (2013)
*Pseudomonas denitrificans*	*Aphanothece halophytica*	0	NA*	1 day	Shkryl et al. (2020)

ON, overnight; NA, not available; DW, dry weight; FW, fresh weight; *—Identified by HPLC-MS.

## Conclusion

The heterologous production of the compatible solute glycine betaine (GB) was successfully achieved in different *Synechocystis*-based chassis. The characterization of these compatible solutes (CS) deficient chassis (Δ*sps*, Δ*ggpS*, and Δ*sps*Δ*ggpS*) revealed that under saline conditions, the carbon fluxes are redirected from the synthesis of glycogen toward the production of CS and extracellular polysaccharides. In fact, the maximum amount of GB was obtained in Δ*ggpS* harboring the GB device, under 3% NaCl (64.29 µmol/gDW). This production level is promising and not far from applications using *E. coli*. Considering that the knowledge generated by the characterization of the CS deficient mutants will allow the identification of potential targets to optimize our chassis, that our GB production is based on sunlight and CO_2_ fixation, and that there is the possibility of using seawater, *Synechocystis* emerges as a feasible photoautotrophic chassis for large-scale heterologous production of GB or other CS.

## Data Availability

The original contributions presented in the study are included in the article/Supplementary Material, further inquiries can be directed to the corresponding author.

## Abbreviations

CPS, capsular polysaccharides; CS, compatible solutes; DW, dry weight; EPS, extracellular polymeric substances; FW, fresh weight; GB, glycine betaine; GG, glucosylglycerol; NMR, nuclear magnetic resonance; RPS, released polysaccharides; RT-qPCR, reverse transcription quantitative PCR.
