# Supplementary material for: Heterologous Production of Glycine Betaine Using Synechocystis sp. PCC 6803-Based Chassis Lacking Native Compatible Solutes
Source: Front Bioeng Biotechnol. 2022 Jan 7;9:821075. doi: 10.3389/fbioe.2021.821075 (PMC8777070; doi:10.3389/fbioe.2021.821075)
Supplement: Supplementary file 1 [file DataSheet2.docx]

**pSEVA351 P*_trc.x.lacO_*::Ahbet**


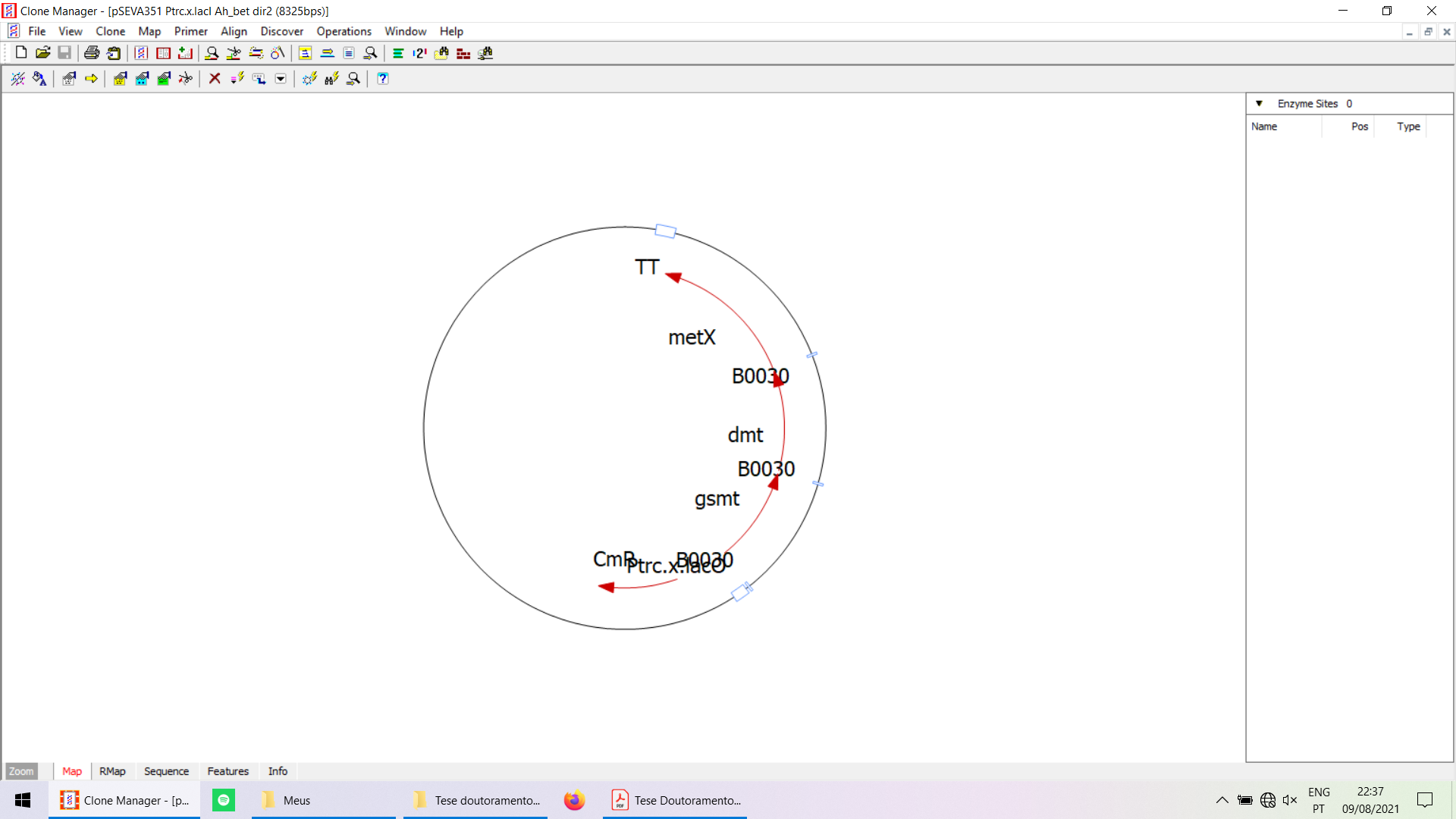


>Ptrc.x.lacO::Ahbet

AATTGTGAGCGCTCACAATTTTCTGAAATGAGCTGTTGACAATTAATCATCCGGCTCGTA

TAATGTGTGGAATTGTGAGCGGATAACAATTTCACACATACTAGAGTACTAGAGATTAAA

GAGGAGAAATACTAGATGGCGATTAAAGAGAAACAGGTGCAAGACTACGGTGAGAATCCC

ATTGAAGTTCGTGACAGCGATCACTATCAAAACGAATACATCGAAGGGTTTGTTGAGAAA

TGGGACGAACTTATTAATTGGCATGCCAGGTCAAGCTCCGAGGGCGAGTTCTTTATTAAG

ACCCTTAAAGAACATGGTGCTAAGCGGGTACTAGATGCGGCCACAGGTACCGGCTTTCAT

TCTATTCGACTAATTGAAGCCGGTTTCGATGTGGCCTCCGTTGATGGGAGCGTGGAAATG

CTGGTTAAAGCCTTTGAGAACGCTACGCGCAAAGACCAGATCCTCCGCACCGTGCACTCC

GACTGGCGTCAAGTTACACGTCATATTCAGGAAAGGTTTGATGCCGTGATTTGTCTAGGG

AATAGTTTTACTCATCTATTTTCCGAGGAAGATCGCCGAAAGACATTAGCTGAGTTCTAT

AGTGTATTGAAGCATGATGGGATTTTGATTCTTGACCAACGCAATTACGATTTGATCTTG

GACGAAGGTTTTAAGAGCAAACATACCTACTACTACTGTGGGGATAATGTAAAGGCCGAA

CCCGAATATGTTGACGATGGTTTGGCGCGCTTTAGATATGAGTTTCCAGATCAAAGCGTA

TATCATCTGAACATGTTTCCCTTGAGGAAGGATTATGTTCGTCGCCTACTGCATGAAGTG

GGTTTCCAGGATATTACGACCTATGGAGATTTTCAAGAAACCTATCACCAAGACGATCCC

GATTTTTATATTCATGTGGCTAAAAAAGATTAATAATACTAGAGATTAAAGAGGAGAAAT

ACTAGATGACAAAAGCCGATGCTGTAGCCAAACAAGCTCAAGACTACTATGATAGTGGAT

CTGCCGATGGATTTTATTACAGGATTTGGGGGGGGGAGGACCTTCACATTGGGATTTATA

ATACCCCCGATGAGCCCATTTATGATGCCTCCGTGCGCACCGTATCAAGGATTTGTGATA

AAATTAAAAACTGGCCCGCCGGAACCAAAGTCCTGGACCTGGGGGCAGGGTATGGTGGCT

CCGCGCGTTATATGGCGAAACATCATGGGTTTGATGTCGATTGCCTAAATATTTCCTTAG

TCCAAAATGAACGGAATCGCCAGATGAATCAAGAACAAGGCCTGGCGGACAAGATCCGGG

TGTTTGATGGGAGCTTTGAGGAACTGCCATTCGAAAATAAATCCTATGATGTGCTTTGGA

GCCAAGATTCCATATTGCATAGTGGCAACCGGCGGAAAGTGATGGAAGAAGCAGATAGGG

TGTTAAAGTCCGGAGGTGATTTTGTTTTTACCGATCCGATGCAAACTGATAACTGCCCCG

AAGGCGTATTGGAGCCTGTTTTAGCTCGAATCCATCTGGATTCTCTCGGCTCTGTCGGAT

TTTACCGGCAAGTGGCCGAGGAACTAGGTTGGGAGTTTGTGGAGTTTGATGAACAAACCC

ACCAACTCGTCAATCATTACAGCCGCGTGCTTCAAGAGCTAGAAGCCCATTATGATCAGT

TGCAACCTGAATGTAGCCAAGAGTACCTAGACCGTATGAAAGTGGGGCTCAATCATTGGA

TCAATGCTGGCAAAAGTGGGTATATGGCCTGGGGTATCTTAAAGTTTCATAAGCCCTAAT

AACCTAGGATTAAAGAGGAGAAATACTAGATGACCGAAGGGCACCCGGATAAAGTATGTG

ATCAAATTAGCGATACAATTTTGGACGCGTTACTGACCCTTGATCCCAATTCCCGCGTTG

CCGCCGAAACAGTCGTTAACACCGGATTAACGTTGGTTACCGGCGAAATTACTTCCCAAG

CCCACATCAACTTTGTAGAGTTGATTCGCCAAAAAATCGCGGAAATTGGTTATACTAATG

CCGATAATGGCTATTCCGCCAACTCCTGTGCGGTTATGTTAGCTATCGACGAGCAAAGTC

CCGATATCTCCCAGGGGGTGACAGCCGCTCAGGAACAGCGTCACGCGTTAAGTGACGACG

AACTGGATAAAATTGGGGCGGGGGATCAAGGTCTGATGTTTGGTTACGCCTGTAATGAGA

CACCGGAGCTAATGCCCCTACCTATTAGTTTGGCCCATAGAATTGCGCTGCGGCTTTCCG

AAGTGCGCAAATCCGGCCAACTAGCGTACCTCAGGCCAGATGGTAAGACCCAAGTCAGTA

TTTTGTACGAAGATGGTTCCCCTGTAGCTATTGATACTATTTTAATCTCCACTCAACATG

ACGAGCACATTGGGGATATTACCGATAACGATGCCGTTCAAGCCAAAATCAAAGCTGATT

TGTGGGACGTGGTAGTCGGGCACTGTTTTTCTGATATTGCCTTGAAGCCTACTGACAAGA

CCCGCTTTATTGTAAACCCAACGGGCAAGTTCGTGGTTGGCGGTCCCCAGGGTGATGCGG

GTCTGACTGGCCGCAAGATTATCGTTGATACCTATGGCGGGTACTCCCGGCATGGCGGGG

GAGCTTTTTCTGGCAAAGATCCTACTAAAGTTGACCGGAGTGCCGCTTACGCCGCCCGTT

ACGTTGCAAAAAACATCGTCGCCGCGGGTTTAGCCGATAAATGTGAAGTCCAAGTATCTT

ATGCCATTGGGGTTGCGCGGCCAGTTTCGGTTTTGATCGATACGTTCGGAACCGGCAAAG

TGGACGAGGAAAAACTCTTGGAAGTGGTCTTGGCCAACTTTGAATTGCGTCCAGCGGGGA

TCATTCAATCTTTGAACCTCCGCAACCTCCCCGCCGAACGCGGGGGTCGTTTCTATCAAG

ATGTGGCCGCGTACGGCCACTTTGGTCGTAATGATCTCGACCTCCCCTGGGAGTACACCG

ACAAAGTTGACGTTTTGAAGGCCGCCTTTGCGTCAAGTCCTCAAGCTGTGGCTGTTTAAT

AACCTAGGCCAGGCATCAAATAAAACGAAAGGCTCAGTCGAAAGACTGGGCCTTTCGTTT

TATCTGTTGTTTGTCGGTGAACGCTCTCTACTAGAGTCACACTGGCTCACCTTCGGGTGG

GCCTTTCTGCGTTTATA
