# Supplementary material for: Heterologous Production of Glycine Betaine Using Synechocystis sp. PCC 6803-Based Chassis Lacking Native Compatible Solutes
Source: Front Bioeng Biotechnol. 2022 Jan 7;9:821075. doi: 10.3389/fbioe.2021.821075 (PMC8777070; doi:10.3389/fbioe.2021.821075)
Supplement: Supplementary file 2 [file DataSheet1.docx]

Supplementary Material

Ferreira *et al.* 2021

**
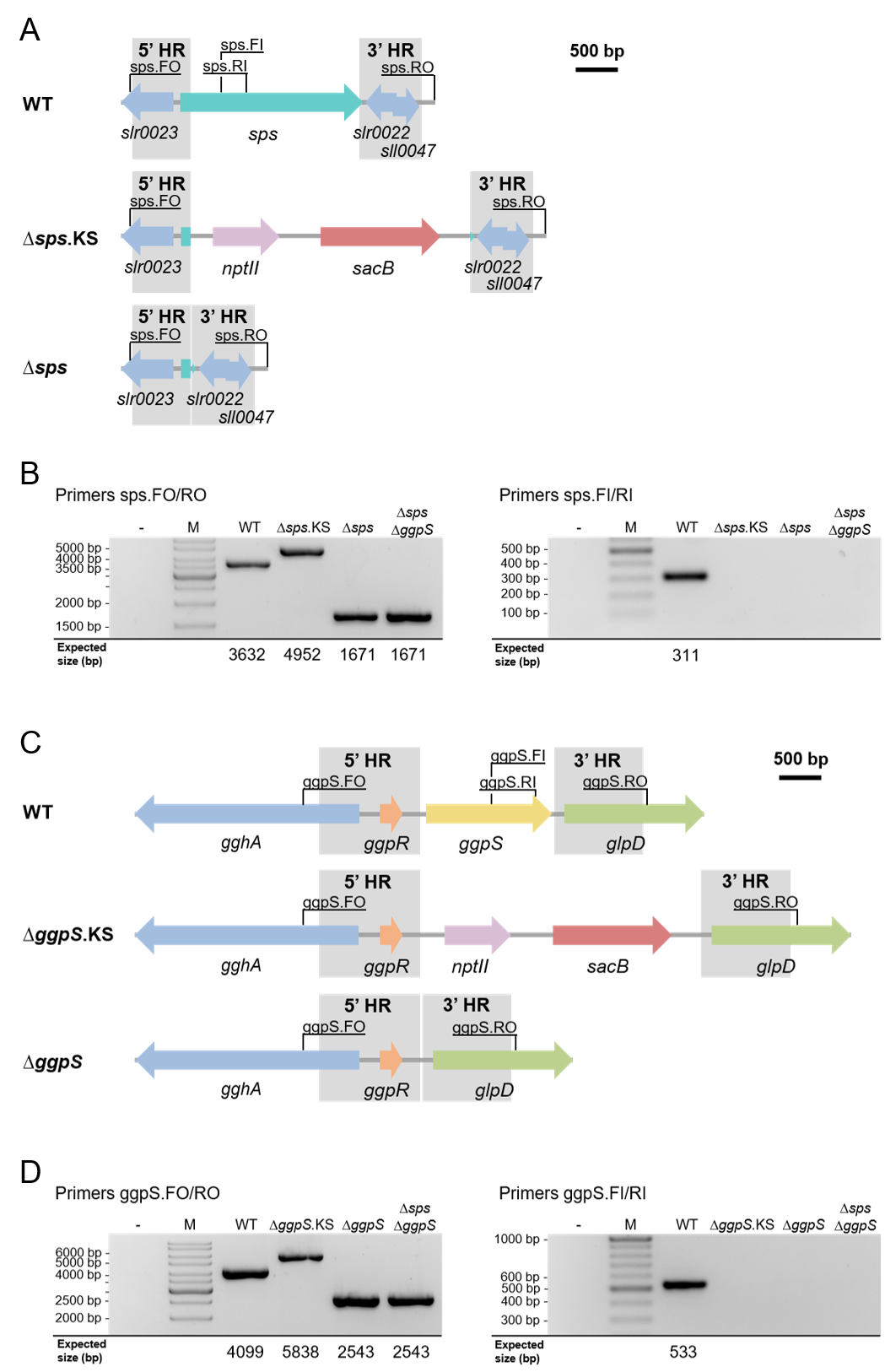
**

**Fig. S1 - Schematic representation of the position of the primers (A and C) and PCR analysis confirming the segregation of the *Synechocystis* ∆*sps*, ∆*ggpS* and ∆*sps*∆*ggpS* mutants (B and D)**. PCR reactions were performed using inner and outer primers for *sps* (sps.FI/RI and sps.FO/RO, respectively) and *ggpS* (ggpS.FI/RI and ggpS.FO/RO, respectively). Primers are listed in Table S2. The expected band sizes are indicated. -, negative control (absence of template); M, molecular marker: GeneRuler DNA Ladder Mix (Thermo Scientific^TM^); WT, wild-type; bp, base pairs.

**
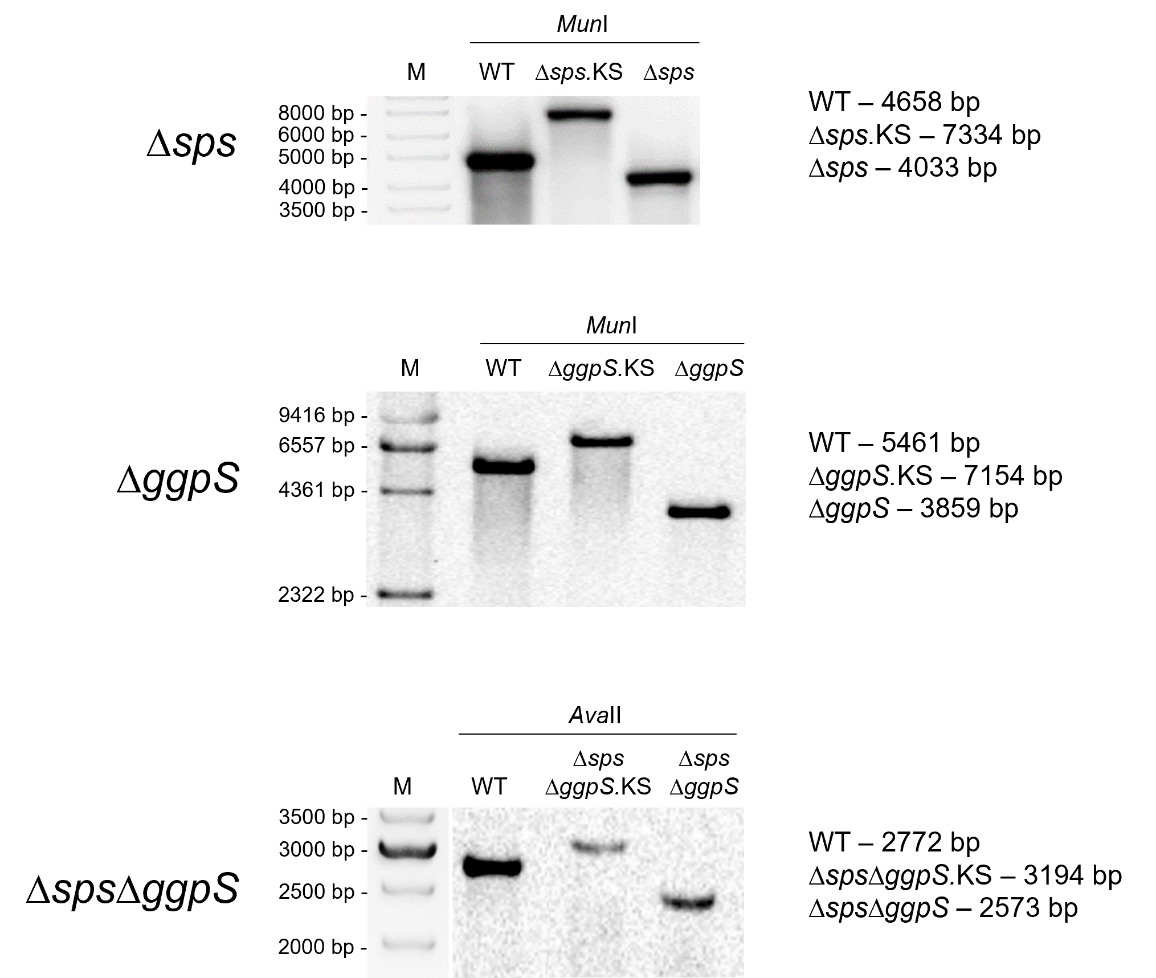
**

**Fig. S2 - Southern blot analysis confirming the segregation of the *Synechocystis* ∆*sps*, ∆*ggpS* and ∆*sps*∆*ggpS* mutants.** The DNA was digested with the endonucleases *Mun*I (WT, ∆*sps*.KS, ∆*sps*, ∆*ggpS*.KS and ∆*ggpS*) and *Ava*II (WT, ∆*sps*∆*ggpS*.KS and ∆*sps*∆*ggpS*). Digoxigenin-labelled probes covering the 3’-flanking region of *sps* and the 5’-flanking region of *ggpS* were used (see the *Materials* section for details). The sizes of the DNA fragments hybridizing with the probe are indicated. M, molecular marker: GeneRuler DNA Ladder Mix (Thermo Scientific^TM^) for ∆*sps* and ∆*sps*∆*ggpS* and lambda DNA/HindIII marker (Thermo Scientific^TM^) for ∆*ggpS*; WT, wild-type; bp, base pairs.

**
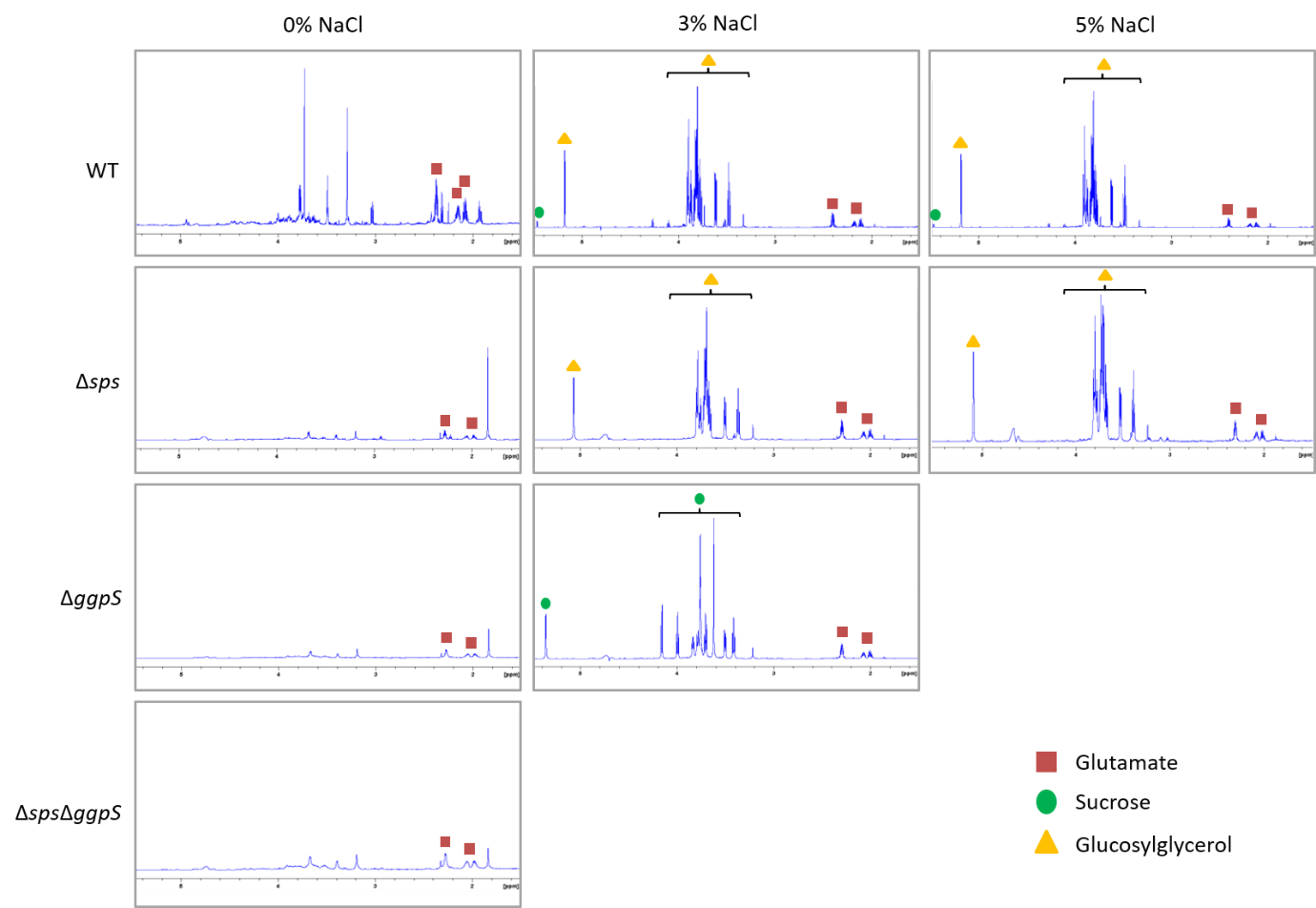
**

**Fig. S3 - Proton nuclear magnetic resonance (NMR) spectra of *Synechocystis* cell-free extracts for the detection of the compatible solutes glutamate, sucrose and glucosylglycerol.** Cell-free extracts were obtained from *Synechocystis* wild-type (WT) and the Δ*sps*, Δ*ggpS* and Δ*sps*Δ*ggpS* mutants, grown in BG11 or BG11 supplemented with 3% or 5% (wt/vol) NaCl (left, middle or right panel, respectively). Spectra were acquired at 25 °C on an AVANCE III 800 spectrometer (Bruker) using a four channel inverse detection probe head with solvent pre-saturation and a recycle delay of 60s to allow full relaxation of the resonances.

**
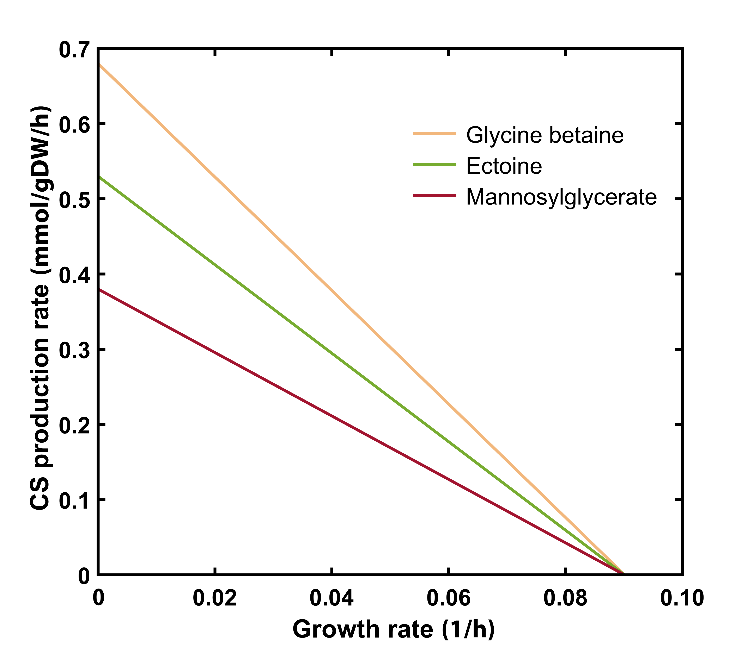
**

**Fig. S4 - Theoretical productivity of the heterologous compatible solutes glycine betaine, ectoine and mannosylglycerate**, as predicted by the updated version of the genome-scale metabolic model *i*Syn811 (Montagud *et al.*, 2011). The lines represent the compatible solute production rate as a function of *Synechocystis* wild-type growth under autotrophic conditions.

**
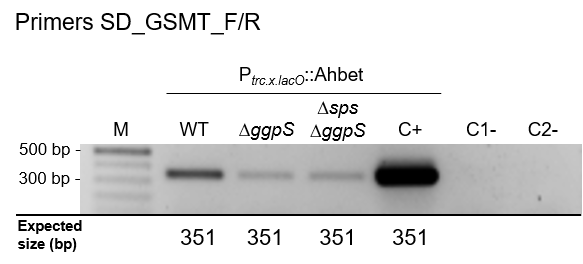
**

**Fig. S5 - PCR analysis confirming the presence of the plasmid containing the glycine betaine synthetic device (pSEVA351 P*_trc.x.lacO_*::Ahbet) in *Synechocystis* wild-type (WT) and ∆*ggpS* and ∆*sps*∆*ggpS* mutants.** PCR reactions were performed using SD_GSMT_F/R (primers listed in Table S2). The expected band sizes are indicated. C+, positive control (plasmid DNA pSEVA351 P*_trc.x.lacO_*::Ahbet); C1-, negative control (*Synechocystis* WT genomic DNA); C2-, negative control (no template); M, molecular marker: GeneRuler DNA Ladder Mix (Thermo Scientific^TM^); bp, base pairs.

**
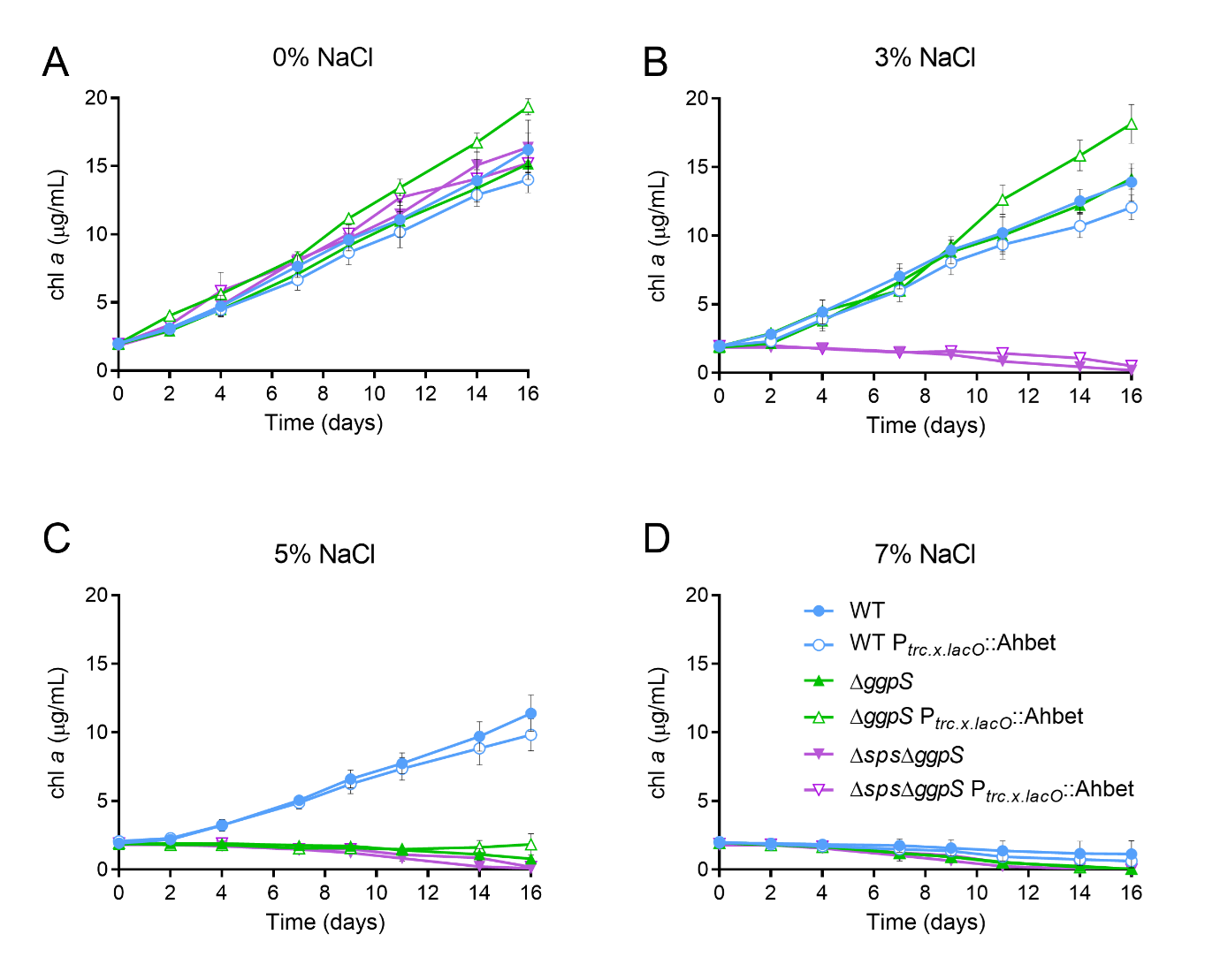
**

**Fig. S6 - Growth curves of *Synechocystis* wild-type (WT), ∆*ggpS,* ∆*sps*∆*ggpS* and the corresponding strains harboring the glycine betaine synthetic device (P*_trc.x.lacO_*::Ahbet).** Cultures were grown in BG11 (**A**) or BG11 supplemented with 3% (**B**), 5% (**C**) or 7% (**D**) (wt/vol) NaCl, at 30 ºC with orbital shaking (150 rpm) under a 12 h light (25 μE/m^2^/s) / 12 h dark regimen. Growth was monitored by measuring chlorophyll *a* (chl *a*) expressed as µg per mL of culture. Error bars correspond to standard deviations from, at least, three biological replicates with technical duplicates.

**
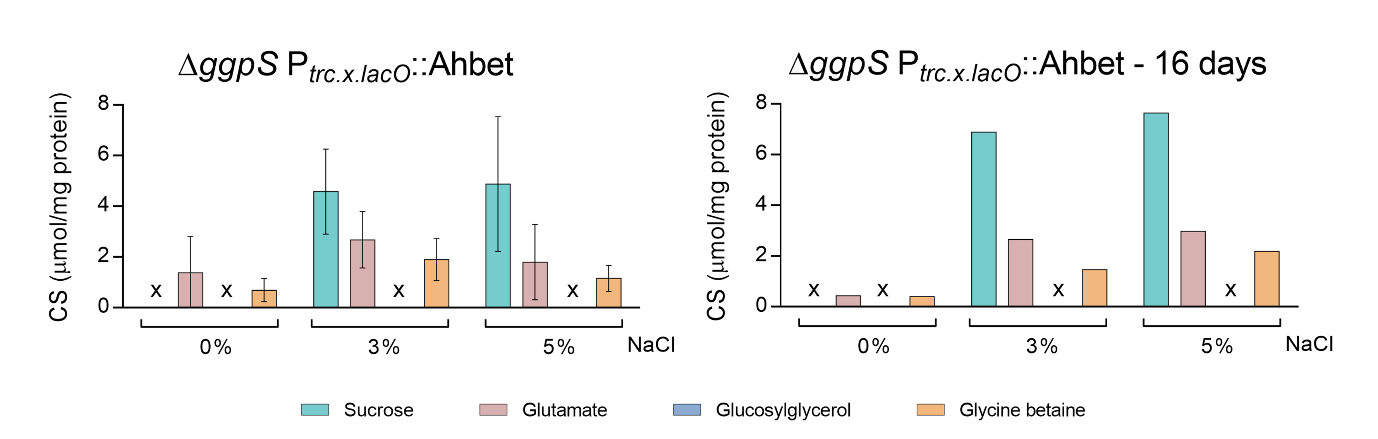
**

**Fig. S7 - Effect of NaCl on the synthesis of native compatible solutes sucrose, glutamate and glucosylglycerol and the heterologous glycine betaine in *Synechocystis* ∆*ggpS* P*_trc.x.lacO_*::Ahbet after 4 days (A) and 16 days (B) of cultivation.** Cultures were grown in BG11 or BG11 supplemented with 3% or 5% (wt/vol) NaCl, at 30 ºC with orbital shaking (150 rpm) under a 12 h light (25 μE/m^2^/s) / 12 h dark regimen. Compatible solutes were quantified by H-NMR, and the results were normalized per mg of protein. x - not detected. Error bars correspond to standard deviations from three biological replicates for 4 days of cultivation and one biological replicate for 16 days of cultivation.

**
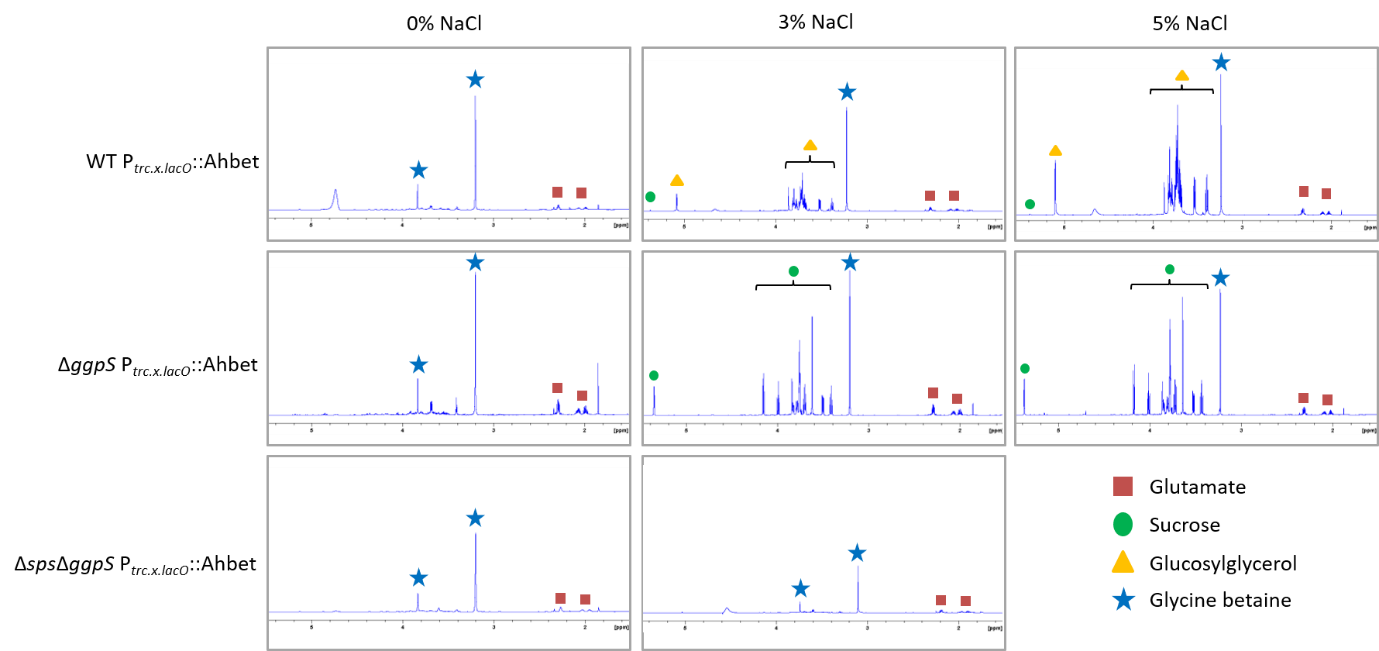
**

**Fig. S8 - Proton NMR spectra of *Synechocystis* cell-free extracts for the detection of the compatible solutes glutamate, sucrose, glucosylglycerol and glycine betaine.** Cell-free extracts were obtained from *Synechocystis* WT P*_trc.x.lacO_*::Ahbet, ∆*ggpS* P*_trc.x.lacO_*::Ahbet and ∆*sps*∆*ggpS* P*_trc.x.lacO_*::Ahbet, grown in BG11 or BG11 supplemented with 3% or 5% (wt/vol) NaCl (left, middle or right panel, respectively). Spectra were acquired at 25 °C on an AVANCE III 800 spectrometer (Bruker) using a four channel inverse detection probe head with solvent pre-saturation and a recycle delay of 60s to allow full relaxation of the resonances.

**Table S1 - Growth decrease (OD_730_) of *Synechocystis* wild-type (WT), Δ*sps*, Δ*ggpS*, Δ*sps*Δ*ggpS* and mutants harboring P*_trc.x.lacO_*::Ahbet in 3%, 5% or 7% (wt/vol) NaCl compared with 0% NaCl at day 16.**

| **Strain** | **Growth decrease^a^ compared with 0% NaCl** | | |
| --- | --- | --- | --- |
|  | **3% NaCl**  **(510 mM)** | **5% NaCl**  **(860 mM)** | **7% NaCl**  **(1200 mM)** |
| **Mutants** | | | |
| **WT** | 22.5% | 43.4% | 85.6% |
| **Δ*sps*** | 28.7% | 55.3% | 88.7% |
| **Δ*ggpS*** | 17.0% | 87.8% | 91.6% |
| **Δ*sps*Δ*ggpS*** | 91.0% | 92.3% | 93.1% |
| **Mutants harboring the glycine betaine device** | | | |
| **WT P*_trc.x.lacO_*::Ahbet** | 31.0% | 43.4% | 89.0% |
| **Δ*ggpS* P*_trc.x.lacO_*::Ahbet** | 17.8% | 81.2% | 92.0% |
| **Δ*sps*Δ*ggpS* P*_trc.x.lacO_*::Ahbet** | 90.3% | 92.0% | 93.3% |

^a^Statistical significance of *P* ≤ 0.0001.

**Table S2 -** **RT-qPCR data of *gsmt*, *dmt* and *metX* relative expression in *Synechocystis* chassis (WT, ∆*ggpS* and ∆*sps*∆*ggpS*) harboring the glycine betaine synthetic device**. RNA was obtained from cells grown in BG11 or BG11 supplemented with 3% and 5% (wt/vol) NaCl, at 30 ºC with orbital shaking (150 rpm) under a 12 h light (25 μE/m^2^/s) / 12 h dark regimen. The ΔCq represents the expression of the target genes relative to WT P*_trc.x.lacO_*::Ahbet 0% NaCl. The data was obtained from three biological replicates and three technical replicates. Statistical analysis was performed by means of One-way ANOVA (*P*-value).

|  | **Target genes** | | | | | | | | |
| --- | --- | --- | --- | --- | --- | --- | --- | --- | --- |
|  | ***gsmt*** | | | ***dmt*** | | | ***metX*** | | |
|  | **Relative expression**  **(ΔCq)** | **SEM (lg)** | ***P*-value** | **Relative expression**  **(ΔCq)** | **SEM (lg)** | ***P*-value** | **Relative expression**  **(ΔCq)** | **SEM (lg)** | ***P*-value** |
| **WT P*_trc.x.lacO_*::Ahbet 0% NaCl** | 1.00 | 0.70 | N.A. | 1.00 | 0.95 | N.A. | 1.00 | 0.97 | N.A. |
| **WT P*_trc.x.lacO_*::Ahbet 3% NaCl** | 0.28 | 0.32 | 0.075 | 0.21 | 0.63 | 0.117 | 0.19 | 0.78 | 0.124 |
| **WT P*_trc.x.lacO_*::Ahbet 5% NaCl** | 1.15 | 0.96 | 0.873 | 0.36 | 1.61 | 0.470 | 0.26 | 1.80 | 0.398 |
| **∆*ggpS* P*_trc.x.lacO_*::Ahbet 0% NaCl** | 0.34 | 0.41 | 0.128 | 0.13 | 0.78 | 0.076 | 0.11 | 0.91 | 0.074 |
| **∆*ggpS* P*_trc.x.lacO_*::Ahbet 3% NaCl** | 0.37 | 0.17 | 0.119 | 0.15 | 0.32 | 0.051 | 0.10 | 0.17 | 0.027 |
| **∆*ggpS* P*_trc.x.lacO_*::Ahbet 5% NaCl** | 0.62 | 0.14 | 0.385 | 0.09 | 1.79 | 0.166 | 0.19 | 0.09 | 0.071 |
| ***∆sps∆ggpS* P*_trc.x.lacO_*::Ahbet 0% NaCl** | 1.46 | 0.44 | 0.543 | 1.02 | 0.71 | 0.985 | 0.79 | 0.86 | 0.807 |
| ***∆sps∆ggpS* P*_trc.x.lacO_*::Ahbet 3% NaCl** | 0.97 | 0.50 | 0.966 | 0.84 | 0.77 | 0.849 | 0.74 | 0.92 | 0.757 |

SEM: Standard Error of the Mean; N.A.: not applicable.

**Table S3 - Quantification of glycine betaine in the extracellular medium used for the cultivation of *Synechocystis* chassis (WT, ∆*ggpS* and ∆*sps*∆*ggpS*) harboring the glycine betaine device.** Glycine betaine was quantified by H-NMR and the results are presented as the average ± standard deviation from three biological replicates, except for the Δ*ggpS* P*_trc.x.lacO_*::Ahbet grown for 16 days (one biological replicate).

| **Mutant** | **Glycine betaine (µM)** | | |
| --- | --- | --- | --- |
|  | **0% NaCl** | **3% NaCl**  **(510 mM)** | **5% NaCl**  **(860 mM)** |
| **WT P*_trc.x.lacO_*::Ahbet** | 2.50 ± 0.12 | 2.69 ± 0.56 | 1.55 ± 0.08 |
| **Δ*ggpS* P*_trc.x.lacO_*::Ahbet** | 0.49 ± 0.12 | 0.24 ± 0.05 | 0.36 ± 0.22 |
| **Δ*sps*Δ*ggpS* P*_trc.x.lacO_*::Ahbet** | 2.20 ± 1.25 | 0.09 ± 0.02 | - |
| **Δ*ggpS* P*_trc.x.lacO_*::Ahbet – 16 days** | 0.72 | 2.12 | 0.18 |

**Table S4 -** **List of primers used in this study.**

| **Primer name** | **Sequence*** | **T_a_ (ºC)**** | **Purpose** | **Reference/Source** |
| --- | --- | --- | --- | --- |
| pUC_F | AGGGTTTTCCCAGTCACGAC | 57 | Amplification of Ahbet synthetic construction | This study |
| pUC_R | ACACAGGAAACAGCTATGAC |  |  |  |
| PS1 | AGGGCGGCGGATTTGTCC | 60 | Confirmation of construct in pSEVA /strains harboring the GB device | Silva-Rocha *et al.* (2013) |
| PS2 | GCGGCAACCGAGCGTTC |  |  |  |
| ggpS.5-O | GCTGGCTCGAGAACACCGTAGGGCAGGGAATAGGTC | 60 | Generation of the Δ*ggpS* mutant / Southern probe | Ferreira *et al.* (2018) |
| ggpS.5-I | GATTACAACCGGTTGTAATCACGGCTAATGCACCCGACTTCCCGGAACCCAAGTTAATTC |  |  |  |
| ggpS.3-O | CTGGCTTTAACCCTGTCGAGGGAACCATCATAG | 60 | Generation of the Δ*ggpS* mutant | Ferreira *et al.* (2018) |
| ggpS.3-I | GATTACAACCGGTTGTAATCGTGGTCGGCGGATGGTAACCAAATAACCATTGTC |  |  |  |
| sps.5-O | CGCCGCTCGAGGCAATGAATTGGGCGGTGGAATAG | 60 | Generation of the Δ*sps* mutant | This study |
| sps.5-I | GATTACACCCGGGTGTAATCAGTTCCAGCACATATTTGGTTTGCCCGCCGGTGTC |  |  |  |
| sps.3-O | AAGGTTTCTCGCCACAATAGGTCAGGCTGGCATAG | 60 | Generation of the Δ*sps* mutant / Southern probe | This study |
| sps.3-I | GATTACACCCGGGTGTAATCCTGGCCCATTACCGCTTCTTTGAGTTGTTAGACCC |  |  |  |
| NeoSacB2F | GCTGGAATTCAGGAAGCGGAACACGTAGAAAG | 60 | Amplification of the *nptII/sacB* cassette | Schafer *et al.* (1994) |
| NeoSacB3R | CTACCAATTGCGTAACAGATGAGGGCAAGCGGATGG |  |  |  |
| Km.KmScFwd | CTGACCCCGGGTGAATGTCAGCTACTGG | 58 | Amplification of the *nptII/sacB* cassette | Pinto *et al.* (2015) |
| KmRev | CAAACCCGGGCGATTTACTTTTCGACCTC |  |  |  |
| sps.FO | TAGATCTTGGGCTTGGTTGAGG | 63 | Confirmation of segregation of ∆*sps* mutant | This study |
| sps.RO | TGGTGAACATCGGCTTGTC |  |  |  |
| sps.FI | CGTCTCCTGCTCAGTGGGATTAAAG | 56 |  |  |
| sps.RI | GGTCGGGACAAAGCGAGGATAATAG |  |  |  |
| ggpS.FO | GACAAATGGCCGCTTCGCTGTCTTC | 63 | Confirmation of segregation of ∆*ggpS* mutant | This study |
| ggpS.RO | CTGCTGGCATCACCCGGTTAGTTTC |  |  |  |
| ggpS.FI | CGTGGGCACCAATCCGGCAAATATC | 56 |  | Ferreira *et al.* (2018) |
| ggpS.RI | GGTTAGTCAACACCGCATCGGGTAG |  |  |  |
| rnpBF1 | CGTTAGGATAGTGCCACAG | 56 | RNA control PCRs | Pinto *et al.* (2012a) |
| rnpBR1 | CGCTCTTACCGCACCTTTG |  |  |  |
|  |  |  |  |  |
| BD16SF1 | CACACTGGGACTGAGACAC | 56 | cDNA control PCRs | Pinto *et al.* (2012b) |
| BD16SR1 | CTGCTGGCACGGAGTTAG |  |  |  |
| SD_GSMT_F | TGCTAAGCGGGTACTAGATGC | 56 | RT-qPCR | This study |
| SD_GSMT_R | CCTTCGTCCAAGATCAAATCG |  |  |  |
| SD_DMT_F | ATTTATGATGCCTCCGTGCG | 56 |  |  |
| SD_DMT_R | GCTTCTTCCATCACTTTCCGC |  |  |  |
| SD_metX_F | TGAGACACCGGAGCTAATGC | 56 |  |  |
| SD_metX_R | AAGCGGGTCTTGTCAGTAGGC |  |  |  |

^*^Restriction enzyme recognition sites are underlined

^**^T_a_ – annealing temperature

**Table S5 - Amplicon sizes and parameters derived from RT-qPCR data analysis.**

| **Gene** | **Amplicon size (bp)** | **Amplicon Tm (°C)** | **NTC* (Cq)** | **Amplification efficiency (E)** | **R^2^** | **Slope** | **y-interception** |
| --- | --- | --- | --- | --- | --- | --- | --- |
| *gsmt* | 351 | 82.0 | N.D. | 89.2 | 0.998 | -3.611 | 23.405 |
| *dmt* | 335 | 83.0 | 72.0** | 92.0 | 0.998 | -3.529 | 21.098 |
| *metX* | 312 | 82.0 | N.D. | 94.5 | 0.998 | -3.462 | 19.014 |

*: no template control, **: primer dimer, N.D.: not detected

**References**

Ferreira, E. A., Pacheco, C. C., Pinto, F., Pereira, J., Lamosa, P., Oliveira, P. *et al.* (2018). Expanding the toolbox for *Synechocystis* sp. PCC 6803: validation of replicative vectors and characterization of a novel set of promoters. *Synth Biol, 3*.

Montagud, A., Zelezniak, A., Navarro, E., de Cordoba, P. F., Urchueguia, J. F., & Patil, K. R. (2011). Flux coupling and transcriptional regulation within the metabolic network of the photosynthetic bacterium *Synechocystis* sp. PCC 6803. *Biotechnol J, 6*, 330-342.

Pinto, F., Pacheco, C. C., Ferreira, D., Moradas-Ferreira, P., & Tamagnini, P. (2012a). Selection of suitable reference genes for RT-qPCR analyses in cyanobacteria. *PLoS One, 7*, e34983.

Pinto, F., Pacheco, C. C., Oliveira, P., Montagud, A., Landels, A., Couto, N. *et al.* (2015). Improving a *Synechocystis*-based photoautotrophic chassis through systematic genome mapping and validation of neutral sites. *DNA Res, 22*, 425-437.

Pinto, F., van Elburg, K. A., Pacheco, C. C., Lopo, M., Noirel, J., Montagud, A. *et al.* (2012b). Construction of a chassis for hydrogen production: physiological and molecular characterization of a *Synechocystis* sp. PCC 6803 mutant lacking a functional bidirectional hydrogenase. *Microbiol, 158*(2), 448-464.

Schafer, A., Tauch, A., Jager, W., Kalinowski, J., Thierbach, G., & Puhler, A. (1994). Small mobilizable multi-purpose cloning vectors derived from the *Escherichia coli* plasmids pK18 and pK19: selection of defined deletions in the chromosome of *Corynebacterium glutamicum*. *Gene, 145*, 69-73.

Silva-Rocha, R., Martínez-García, E., Calles, B., Chavarría, M., Arce-Rodríguez, A., de las Heras, A. *et al.* (2013). The Standard European Vector Architecture (SEVA): a coherent platform for the analysis and deployment of complex prokaryotic phenotypes. *Nucleic Acids Res, 41*, D666-D675.
